# Vγ4^+^γδT Cells Aggravate Severe H1N1 Influenza Virus Infection-Induced Acute Pulmonary Immunopathological Injury *via* Secreting Interleukin-17A

**DOI:** 10.3389/fimmu.2017.01054

**Published:** 2017-08-31

**Authors:** Chunxue Xue, Mingjie Wen, Linlin Bao, Hui Li, Fengdi Li, Meng Liu, Qi Lv, Yunqing An, Xulong Zhang, Bin Cao

**Affiliations:** ^1^Department of Respiratory and Critical Care Medicine, Beijing Luhe Hospital, Capital Medical University, Beijing, China; ^2^Department of Immunology, The Research Centre of Microbiome, School of Basic Medical Sciences, Capital Medical University, Beijing, China; ^3^Institute of Laboratory Animal Sciences, Chinese Academy of Medical Sciences, Beijing, China; ^4^Department of Pulmonary and Critical Care Medicine, China-Japan Friendship Hospital, Beijing, China; ^5^Department of Pulmonary and Critical Care Medicine, Beijing Hospital of Traditional Chinese Medicine Affiliated to Capital Medical University, Beijing, China; ^6^Center for Respiratory Diseases, Department of Pulmonary and Critical Care Medicine, China-Japan Friendship Hospital, National Clinical Research Center for Respiratory Diseases, Beijing, China; ^7^Department of Respiratory Medicine, Capital Medical University, Beijing, China

**Keywords:** A (H1N1) pdm09 virus, influenza, γδT cell subsets, acute lung injury, interleukin-17A, NKG2D

## Abstract

The influenza A (H1N1) pdm09 virus remains a critical global health concern and causes high levels of morbidity and mortality. Severe acute lung injury (ALI) and acute respiratory distress syndrome (ARDS) are the major outcomes among severely infected patients. Our previous study found that interleukin (IL)-17A production by humans or mice infected with influenza A (H1N1) pdm09 substantially contributes to ALI and subsequent morbidity and mortality. However, the cell types responsible for IL-17A production during the early stage of severe influenza A (H1N1) pdm09 infection remained unknown. In this study, a mouse model of severe influenza A (H1N1) pdm09 infection was established. Our results show that, in the lungs of infected mice, the percentage of γδT cells, but not the percentages of CD4^+^Th and CD8^+^Tc cells, gradually increased and peaked at 3 days post-infection (dpi). Further analysis revealed that the Vγ4^+^γδT subset, but not the Vγ1^+^γδT subset, was significantly increased among the γδT cells. At 3 dpi, the virus induced significant increases in IL-17A in the bronchoalveolar lavage fluid (BALF) and serum. IL-17A was predominantly secreted by γδT cells (especially the Vγ4^+^γδT subset), but not CD4^+^Th and CD8^+^Tc cells at the early stage of infection, and IL-1β and/or IL-23 were sufficient to induce IL-17A production by γδT cells. In addition to secreting IL-17A, γδT cells secreted interferon (IFN)-γ and expressed both an activation-associated molecule, natural killer group 2, member D (NKG2D), and an apoptosis-associated molecule, FasL. Depletion of γδT cells or the Vγ4^+^γδT subset significantly rescued the virus-induced weight loss and improved the survival rate by decreasing IL-17A secretion and reducing immunopathological injury. This study demonstrated that, by secreting IL-17A, lung Vγ4^+^γδT cells, at least, in part mediated influenza A (H1N1) pdm09-induced immunopathological injury. This mechanism might serve as a promising new target for the prevention and treatment of ALI induced by influenza A (H1N1) pdm09.

## Introduction

The influenza A (H1N1) pdm09 virus originated in Mexico and the southwest of the United States, and it remains a critical global health concern and causes high levels of mortality (1–3). About half of the deaths and critical illnesses caused by the virus have occurred among healthy young or middle-aged people. The clinical manifestations among severely infected patients are edema of the trachea, bronchial and alveoli mucosa, diffuse alveolar injury, substantial inflammatory cell infiltration, and hypoxemia. Severe acute lung injury (ALI) and acute respiratory distress syndrome (ARDS) are the major outcomes among severely infected patients and those who die from the infection (4, 5).

“Cytokine storms” play an important role in lung immunopathological injury (6–8). Among the cytokines, the proinflammatory interleukin-17 (IL-17) family are important cytokines in mucosal immunity, as they can activate and recruit various immune cells (9). IL-17A can also participate in various types of pathogen-induced immunopathological organ damage, including lung damage (10, 11). Our previous report showed that early-stage neutralization of IL-17A by monoclonal antibodies (mAbs) in mice infected with influenza A (H1N1) pdm09 virus relieved lung tissue injury and prolonged survival time (12). Crowe et al. also found that IL-17RA-deficient mice exhibit less weight loss and an improved survival rate compared to wild-type control mice when infected with influenza virus (13). However, the major source of IL-17A during the early stage of severe influenza A (H1N1) pdm09 infection has not been fully elucidated.

Many innate and adaptive immune cells can secret IL-17A, such as γδT cells (14), natural killer T (NKT) cells (15), IL-17-producing CD4^+^ T cells (Th17), and IL-17-producing CD8^+^ T cells (Tc17) (16–18). The lungs are unique organs in that they are rich in innate immune cells. Unlike conventional αβT cells, γδT cells are found in abundance in the epithelial layers of internal tissues such as the lungs and intestines, where they function as a first line of defense (19, 20). γδT cells are unique and distinct from other lymphocyte subsets, such as natural killer (NK), B, and αβT cells, in that they combine adaptive features with rapid, innate-like responses that allow them to play an important role in all the phases of an immune response (21). To mediate the host innate immune response and promote the accumulation of inflammatory cells, γδT cells can rapidly secrete IL-17A much earlier than adaptive Th17 or Tc17 cells (11).

In mice infected with *Mycobacterium tuberculosis*, IL-17A was mainly secreted by lung γδT cells and not Th17 cells (22). In the liver of mice infected with *Listeria monocytogenes*, γδT cells released a large amount of IL-17A within 1 h post-infection, which was significantly larger than the amount of IL-17A secreted by Th17 cells at the late stage of the infection (23). In addition to IL-17A, γδT cells have emerged as the source of other proinflammatory cytokines, such as interferon (IFN)-γ, in multiple models of infection. The secretion of IL-17A and IFN-γ was distinguished on the basis of their expression of the C-C chemokine receptor 6 (CCR6) versus the expression of the costimulatory receptor CD27, respectively (24–27). It has also been reported that human Vγ9Vδ2 T cells can recognize and efficiently kill human or avian influenza viruses infecting monocyte-derived macrophages. The cytotoxicity of Vγ9Vδ2 T cells was dependent on natural killer group 2, member D (NKG2D) activation and was mediated by the Fas–FasL and perforin–granzyme B pathways (27–30). The expression of the intracellular proinflammatory cytokine (IFN-γ), surface activation marker (NKG2D), and cytotoxicity molecule (FasL) on pulmonary γδT cells after influenza A (H1N1) pdm09 virus infection needs to be further investigated.

Murine γδT cells consist of various subsets characterized by their distinct anatomical locations and functional properties (25). The adult mouse peripheral γδT cells mainly comprise the Vγ1^+^γδT and Vγ4^+^γδT subsets, which may exert distinct immune response in different infectious disease models. Vγ4^+^γδT lymphocytes represent one of the major subsets that produce IL-17A in different experimental animal models, such as mycobacterial infection (31), *L. monocytogenes* infection (23), collagen-induced arthritis (32), ovalbumin-induced allergic airway inflammation and airway hyperreactivity (33), and *Staphylococcus aureus* infection (34). By secreting IL-17A, Vγ4^+^γδT cells may increase susceptibility of myocarditis induced by Coxsackie virus, but Vγ1^+^γδT cells exhibit the opposite result (35). However, the dynamics and potential immunopathological mechanisms of γδT cells (and particularly the Vγ1^+^γδT and Vγ4^+^γδT subsets) during the early phase of influenza A (H1N1) pdm09 virus infection need to be further investigated using a mouse model of a severe infection.

In this study, influenza A (H1N1) pdm09 virus induced a significant increase in γδT cells in the lungs of mice at 3 days post-infection (dpi). γδT cells, especially the Vγ4^+^γδT subset, were the main source of IL-17A during the early phase of the infection. Depletion of γδT cells or the Vγ4^+^γδT subset but not the Vγ1^+^γδT subset significantly improved the survival rate and relieved immunopathological injury by reducing the IL-17A secretion. Vγ4^+^γδT cells are a promising immunotherapy target for the prevention and treatment of ALI induced by influenza A (H1N1) pdm09.

## Materials and Methods

### Animals and Virus Strains

Specific pathogen-free, 4–6-week-old female Balb/c mice and influenza A virus strain A/California/07/2009 (H1N1v) were provided by the Institute of Laboratory Animal Science, Peking Union Medical College, China. The experiments were performed in biosafety level 3 facilities in compliance with governmental and institutional guidelines. This study was carried out in accordance with the recommendations of the Chinese National Guidelines for the Care of Laboratory Animals and the Institutional Animal Care and Use Committee of the Institute of Laboratory Animal Science, Peking Union Medical College. The protocol was approved by the Institutional Animal Care and Use Committee (ILAS-PC-2015-016).

### Mouse Model of Severe Influenza A (H1N1) pdm09 Virus Infection

Mice were anesthetized and inoculated intranasally either with virus (10^2^ 50% tissue culture infective dose [TCID_50_] in 50 µl solution per mice) or, in the control group, an equal quantity of phosphate-buffered saline (PBS). The symptom, body weight, and survival rate of the mice were observed daily.

### Hematoxylin and Eosin (H&E) Staining

For each mouse, the whole right lung was fixed in 10% formalin for 24 h and then embedded in paraffin for histological examination. The lung tissue sections (4 µm) were deparaffinized and hydrated using xylene and an alcohol gradient and, then, stained with H&E. The histopathology of the lung tissue was observed by light microscopy.

### Virus Titrations

For each mouse, the whole left lung homogenates were used for virus titration tests using endpoint titration in Madin–Darby canine kidney (MDCK) cells, as described previously (36).

### Isolation of Lung and Spleen Lymphocytes

The lungs were cut up and subsequently digested in Dulbecco’s Modified Eagle Medium (DMEM) (Gibco, Life Technologies, New York) containing 0.1% collagenase I (Gibco, Life Technologies, New York) at 37°C for 60 min. The tissue suspension after digestion was filtered through a 75-µm strainer and then washed with DMEM. The total lung lymphocytes were centrifuged by density gradient centrifugation using 40% Percoll and 70% Percoll (GE Healthcare, Amersham, UK). The lung lymphocytes were collected from the interface between the 40% and 70% Percoll. The lymphocytes were washed two times with PBS and then suspended in PBS. The spleens were disrupted using tissue grinders with a 75-µm nylon strainer, and the cell suspensions were lysed using lysing buffer (BD Pharm Lyse™, New Jersey). The number of cells in each suspension was adjusted to 2.0 × 10^6^ per tube.

### Collection of Bronchoalveolar Lavage Fluid (BALF)

After stripping the fat and connective tissue around the trachea, the exposed trachea was slowly injected into 2 ml PBS. The PBS was then recovered after 1 min and centrifuged at 1,500 rpm for 10 min at 4°C. The supernatant was collected and stored at −80°C.

### Collection of Lung Homogenate Supernatant

Lung homogenates were prepared by homogenizing perfused whole lung tissue using an electric homogenizer for 2 min 30 s in 1 ml PBS. The homogenates were centrifuged at 3,000 rpm for 10 min at 4°C. The supernatant was collected and stored at −80°C.

### Flow Cytometry Analysis

The lymphocytes were stimulated with leukocyte activation cocktail with BD GolgiPlug™ (BD Pharmingen San Diego, CA, USA) for 4 h at 37°C and 5% CO_2_ in an incubator. The cells were pre-incubated with purified rat anti-mouse CD16/CD32 (Mouse BD Fc Block™, 2.4G2, BD Pharmingen™) on ice for 10 min.

For the extracellular cell marker analysis, the cells were incubated with the following fluorescein-conjugated antibodies for 30 min: BV510-anti-CD3ε (145-2C11; BD Biosciences), APC-Cy7-anti-CD3ε (145-2C11; BD Biosciences), PE-Cy7-T-cell receptor (TCR)β (H57-597; BD Biosciences), PE-anti-CD4 (RM4-5; BD Biosciences) PerCP-Cy5.5-anti-CD8a (53-6.7; BD Biosciences), APC-anti-TCR γ/δ (GL3; Biolegend, CA, USA), FITC-anti-TCR Vγ1 (2.11; Biolegend), PE-anti-TCR Vγ4 (UC3, Biolegend), PerCP-efluor710-anti-FasL (MFL3; eBioscience), and PE-Cy7-anti-NKG2D (CX5; eBioscience).

For intracellular cytokine staining, the extracellularly stained cells were fixed and permeabilized using BD Cytofix/Cytoperm™ and BD Perm/Wash™ (BD Biosciences) and, then, incubated with the following fluorescein-conjugated antibodies: PE-Cy7-anti-IFN-γ (XMG1.2; eBioscience) and PE-Cy7-anti-IL-17A (17B7; eBioscience). Finally, samples were acquired using the fluorescence-activated cell sorting (FACS) Canto II system (BD Biosciences). The data were analyzed using a Kaluza analysis with Flowjo 6.1 software.

### Depletion of γδT Cells and the Vγ4^+^γδT and Vγ1^+^γδT Subsets

For γδT cell depletion, two mAbs with specificity for different TCR-γ/δ epitopes were used to maximize γδT cell depletion as previously described (37); 125 µg anti-mouse TCR γ/δ mAbs (GL3; Biolegend) and 125 µg anti-mouse TCR γ/δ mAbs (UC7, Biolegend) were injected intraperitoneally (i.p.) 1 day before infection. For the Vγ1^+^γδT and Vγ4^+^γδT subsets depletion, 250 µg anti-mouse TCR Vγ1 mAbs (2.11; Sungene, Tianjin, China) and anti-mouse TCR Vγ4 mAbs (UC3-10A6; Sungene), respectively, were injected i.p. both 3 and 1 days before infection, respectively. Hamster IgG (HTK888, Biolegend) was used as isotype control. Control mice were injected i.p. with 250 µl PBS or hamster IgG 1 day before infection. Flow cytometry was used to evaluate the depletion of γδT cells and the Vγ1^+^γδT and Vγ4^+^γδT subsets, as previously described in detail (for γδT cell depletion) (38).

### Cytokine Assays

A multiplex biometric immunoassay was used to measure the cytokines in the serum and BALF according to the manufacturer’s instructions [ProcartaPlex Mouse Cytokine & Chemokine Panel 1 (13 plex); eBioscience]. The levels of cytokines were determined using a multiplex array reader from the Luminex™ Instrumentation System (Bio-Plex Workstation; Bio-Rad Laboratories, San Diego, CA, USA). The concentration was calculated using Bio-Plex Manager Software provided by the manufacturer (39).

### Bicinchoninic Acid (BCA) Assay and Lactate Dehydrogenase (LDH) Assay

Bicinchoninic acid protein assay kit (Beyotime Biotechnology, Jiangsu, China) was used to determine the total protein concentration in the BALF. Briefly, 20 µl BALF and 200 µl BCA were mixed in the wells of a 96-well plate. The microplate was incubated at 37°C for 30 min. The optical density at 562 nm (OD_562_) was measured using a microplate reader (Thermo Lab systems Multiskan MK3, Finland). An automatic biochemical analyzer was used to determine LDH activity (InTec PRODUCTS, Inc., Xiamen, China) in the BALF following the manufacturer’s protocol.

### T-Cell Subsets Sorting and Culture

γδT cells, CD4^+^T cells, and CD8^+^T cells were purified from the lungs of wild-type mice or infected mice using mouse TCRγ/δ^+^T cell isolation kit, mouse CD4 (L3T4) MicroBeads, and CD8a (Ly-2) MicroBeads (Miltenyi Biotechnology, Germany) according to the manufacturer’s instructions, respectively. The cell purity reached to 95% as determined by flow cytometry. Purified γδT cells, CD4^+^T cells, and CD8^+^T cells were stimulated by virus and cultured with IL-1β (10 ng/ml) and/or IL-23 (10 ng/ml) for 36 h. The concentrations of IL-17A in cultured supernatants were determined using an enzyme-linked immunosorbent assay (ELISA) (R&D, MN, USA).

### Statistical Analyses

The data are presented as mean ± SEMs. Analysis of variance (ANOVA) was used for analysis of the differences between three or more groups and *t* tests were used for analysis of the differences between two groups. Statistical graphs were obtained using GraphPad Prism 5 software. The *p* value of the difference in survival was determined using the Kaplan–Meier log-rank test. Differences were considered statistically significant when *p* < 0.05 and highly statistically significant when *p* < 0.01 and *p* < 0.001.

## Results

### Influenza A (H1N1) pdm09 Virus Infection Recruited Vγ4^+^γδT Cells to the Lungs

**Figure 1:**
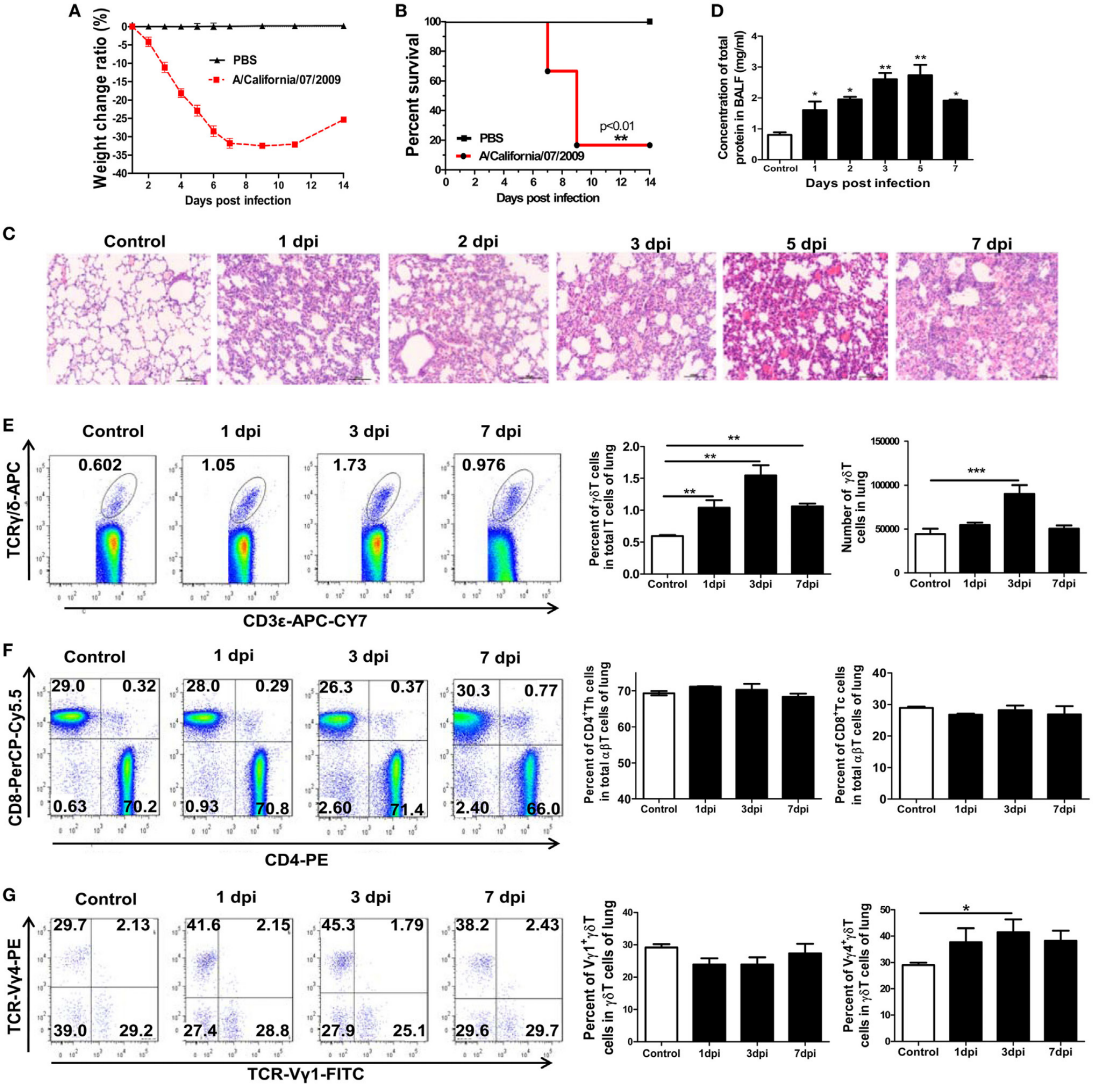
Establishment of a mouse model of severe influenza A (H1N1) pdm09 infection and the changes in T-cell subsets in the lungs after infection. Weight change ratio **(A)** and survival rate **(B)** of infected mice (*n* = 6) and uninfected control mice (*n* = 6) observed over 14 days. **(C)** Immunopathological damage detected by hematoxylin and eosin staining of the lungs of infected mice at the indicated dpi. Original magnification: ×200. **(D)** Changes in total protein concentration in bronchoalveolar lavage fluid (BALF) of infected mice assayed at the indicated dpi. **(E)** Percentage and number of CD3^+^TCRγδ^+^ T cells in the lungs detected using flow cytometry for the control, 1, 3, and 7 dpi groups. **(F)** Percentage of CD3^+^TCRαβ^+^CD4^+^ Th and CD3^+^TCRαβ^+^CD8^+^ Tc cells in the lungs was analyzed using flow cytometry for the control, 1, 3, and 7 dpi groups. The stained lung lymphocytes were first gated on CD3^+^TCRαβ^+^, and then, the CD4^+^Th and CD8^+^Tc subsets were analyzed. **(G)** Percentage of Vγ1^+^γδT and Vγ4^+^γδT cells in the lungs of infected mice at the indicated dpi. The stained lung lymphocytes were first gated on CD3^+^TCRγδ^+^ cells, and then, the Vγ1^+^γδT and Vγ4^+^γδT subsets were analyzed. The results are representative of two independent analyses (*n* = 3 for each group). The numbers on the charts represent the ratio of indicated subsets. *p < 0.05, **p < 0.01, ***p < 0.001.

To further evaluate the roles of γδT cells during the early stage of influenza A (H1N1) pdm09 infection, a mouse model of severe infection was established based on our previous study (40). After infection with the virus, the mice exhibited piloerection, listlessness and loss of appetite. The body weight gradually decreased by 32.48% (Figure 1A), and the mortality was 83.33% at 9 dpi (Figure 1B). Compared to uninfected control mice, in the lungs of infected mice, alveolar space collapse and gradually aggravated histopathological injury were observed and there was a high degree of inflammatory cell infiltration in the lung tissues (Figure 1C). Meanwhile, the concentration of total protein and activity of LDH in the BALF, which represented the pathological damage to the lungs, significantly increased from 1 dpi and peaked on 5 and 3 dpi, respectively (Figure 1D; Figure S1 in Supplementary Material).

The changes in the levels of γδT cells in the lungs of infected or uninfected control mice at multiple time points were analyzed using flow cytometry. As shown in Figure 1E, the percentage of γδT cells among the lung CD3^+^ T lymphocytes in the control mice was 0.59%, but it was significantly raised to 1.04, 1.54, and 1.06% at 1, 3, and 7 dpi, respectively, in the infected mice. Correspondingly, the absolute number of γδT cells also increased significantly from 4.43 × 10^4^ per lung in the control mice to 9.0 × 10^4^ per lung at 3 dpi in the infected mice. Unlike αβT cells, γδT cells leave the thymus as mature cells and the differentiation of functional subtypes of γδT cells is largely preprogrammed in the thymus during the fetal stage (41).

We next investigated whether the accumulated γδT cells in the lungs were recruited from the spleen or peripheral blood. As shown in Figure S2A in Supplementary Material, the percentage of γδT lymphocytes in the peripheral blood was slightly raised at 1 dpi and then reduced to half of the normal level at 3 and 7 dpi. The percentage of γδT lymphocytes in the spleen gradually decreased from 0.62% in the control mice to 0.47, 0.41, and 0.27% at 1, 3, and 7 dpi in the infected mice, respectively. The absolute number of γδT cells also reduced by 44.4 and 88.5% at 3 and 7 dpi, respectively (Figure S2B in Supplementary Material). These data suggest that the spleen might serve as the original source of γδT cells upon pulmonary infection. In addition to γδT lymphocytes, the CD3^+^αβTCR^+^T lymphocytes, and the CD3^+^αβTCR^+^CD4^+^T and CD3^+^αβTCR^+^CD8^+^T subsets, were analyzed. As shown in Figure 1F, there were no clear differences in the percentage and number of CD4^+^T and CD8^+^T cells among the CD3^+^αβTCR^+^T lymphocytes in the infected lungs compared with the control lungs.

Individual γδT-cell subsets are often associated with tissue-specific homing and functions (34, 42). The Vγ1^+^γδT and Vγ4^+^γδT subsets in the lungs and spleens after infection were further investigated. As shown in Figure 1G, the percentage of Vγ4^+^γδT cells gradually increased after infection, and the proportion of Vγ4^+^γδT cells among the γδT cells in the lungs significantly increased from 28.97% in the control mice to 41.48% in the infected mice at 3 dpi. However, the percentage of Vγ1^+^γδT cells slightly declined, with the proportion of Vγ1^+^γδT cells among the γδT cells in the lungs slightly declining from 29.15% in the control mice to 23.87% in the infected mice at 3 dpi. However, the percentage of Vγ4^+^γδT cells in the spleens decreased slightly after infection and the percentage of Vγ1^+^γδT cells showed no obvious change (Figure S2C in Supplementary Material). These results indicate that γδT lymphocytes, especially the Vγ4^+^γδT subset, quickly infiltrated into the lungs at the early stage of the severe influenza A infection, indicating that they have immune protective or immunopathological injury roles.

### Vγ4^+^γδT Cells Were the Major Source of the Elevated IL-17A

**Figure 2:**
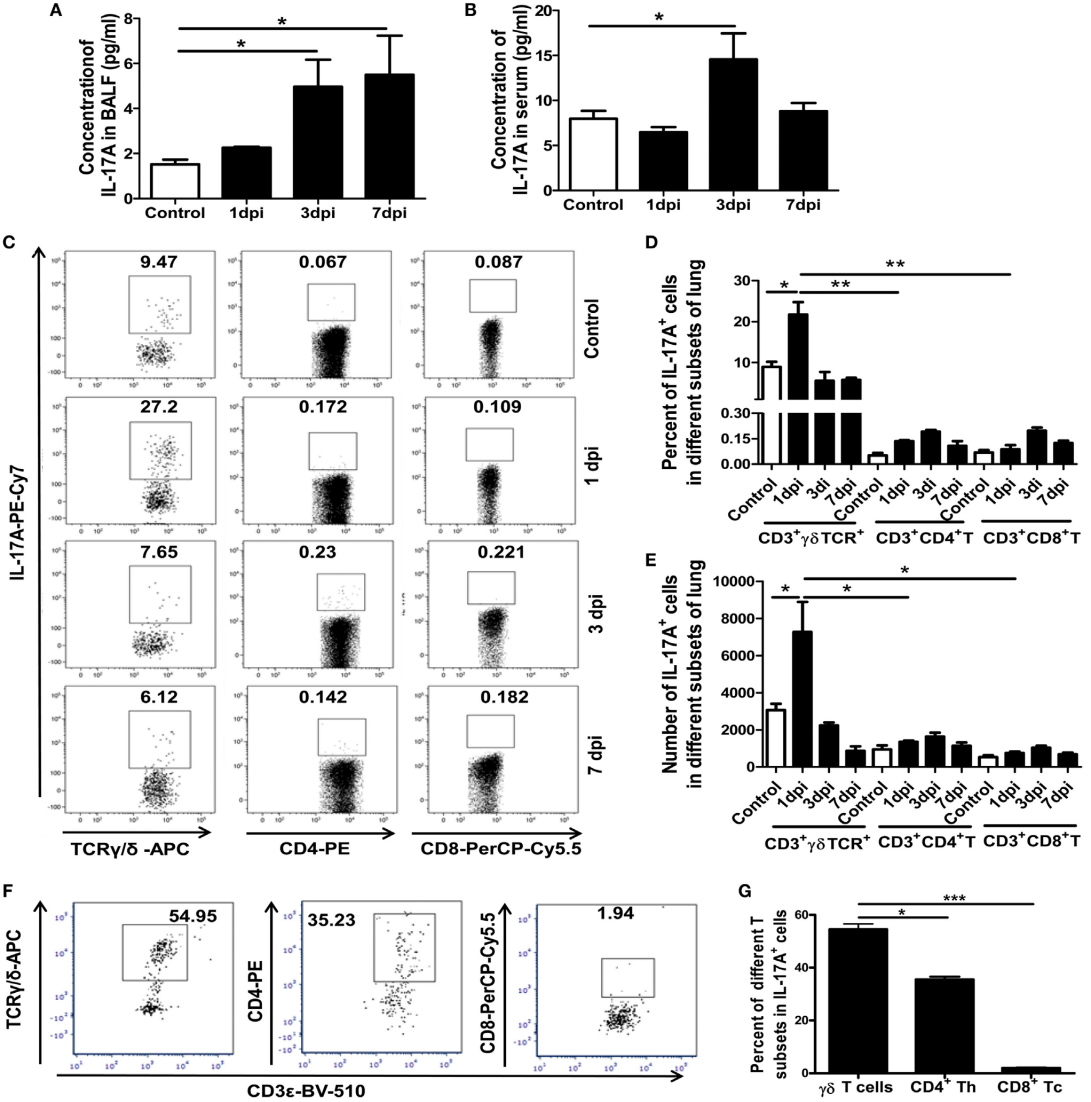
Concentrations of IL-17A in BALF and serum, and proportion and number of γδT17, Th17, and Tc17 cells at different dpi. IL-17A concentrations in BALF **(A)** and serum **(B)** of the control or infected mice at the indicated dpi were quantified using ELISA. **(C)** Expression of IL-17A among CD3^+^TCRγδ^+^, CD3^+^TCRαβ^+^CD4^+^Th, and CD3^+^TCRαβ^+^CD8^+^Tc subsets in the control and infected lungs at the indicated dpi. The numbers represent the proportion of IL-17A-positive cells among different subsets at the indicated dpi. Histogram showing the changes in the proportion **(D)** and number **(E)** of γδT17, Th17, and Tc17 cells at different dpi. **(F)** Lung IL-17A^+^ lymphocytes were first gated, and then, the surface markers for T-cell subtypes were analyzed at 1 dpi. **(G)** Histogram showing the percentage of γδT, CD4^+^Th, and CD8^+^Tc cells among the total lung IL-17A^+^ lymphocytes at 1 dpi. The results are representative of two independent analyses (*n* = 3 for each group). *p < 0.05, **p < 0.01, ***p < 0.001.

Interleukin-17 is a solid link between the innate and adaptive immune responses and can exert both beneficial and deleterious effects. Our previous study reported on the pivotal roles of the proinflammatory cytokine IL-17A in immunopathological lung damage during severe influenza virus infection (12). The concentrations of IL-17A in the BALF and serum of the control and infected mice were detected. As shown in Figure 2A, the concentrations of IL-17A in the BALF significantly increased in the infected mice by 3.26- and 3.61-fold at 3 and 7 dpi, respectively. Meanwhile, the concentration of IL-17A in the serum was also significantly increased (1.84-fold) in the infected mice at 3 dpi (Figure 2B).

The proportion and number of IL-17A-secreting cells, including IL-17-producing γδT cells (γδT17), Th17 cells, and Tc17 cells, in the lungs were assayed using flow cytometry. As shown in Figures 2C–E, γδT cells, Th cells, and Tc cells could secret IL-17A in the uninfected and infected lungs, but the vast majority of the IL-17A^+^ cells were γδT cells. The percentage of γδT17 cells among the γδT cells was significantly increased from 8.97% in the uninfected mice to 21.73% in the infected mice at 1 dpi and returned to the normal level at 3 and 7 dpi. The percentages of Th17 and Tc17 cells were also obviously increased at 1, 3, and 7 dpi. However, the proportion of Th17 and Tc17 among the CD4^+^ or CD8^+^ cells was significantly lower than the proportion of γδT17 cells among the γδT cells (Figures 2C,D).

Consistent with this, the absolute number of γδT17 cells was also significantly increased from 2.74 × 10^3^ in the uninfected lungs to 6.15 × 10^3^ in the infected lungs at 1 dpi. The number of γδT17 cells (6.15 × 10^3^ cells/lung) was also far greater than the number of Th17 cells (1.35 × 10^3^ cells/lung) and Tc17 cells (0.75 × 10^3^ cells/lung) at 1 dpi and, then, gradually decreased at 3 and 7 dpi. However, the number of Th17 and Tc17 gradually increased as the infection progressed and reached a similar level to the number of γδT17 cells at 7 dpi (Figure 2E).

To accurately support our conclusion that γδT cells were the principal producers of IL-17A, IL-17A^+^ cells among the lung lymphocytes were first gated and, then, surface markers for T-cell subtypes were analyzed. As shown in Figures 2F,G, at 1 dpi, about 56, 36, and 2% of the IL-17A^+^ cells were γδT, CD4^+^Th, and CD8^+^Tc cells, respectively. These results further proved that γδT cells were the major source of IL-17A in the lungs at the early stage of severe infection with influenza A (H1N1) pdm09, which indicated that γδT cells might be involved in lung immunopathological injury.

**Figure 3:**
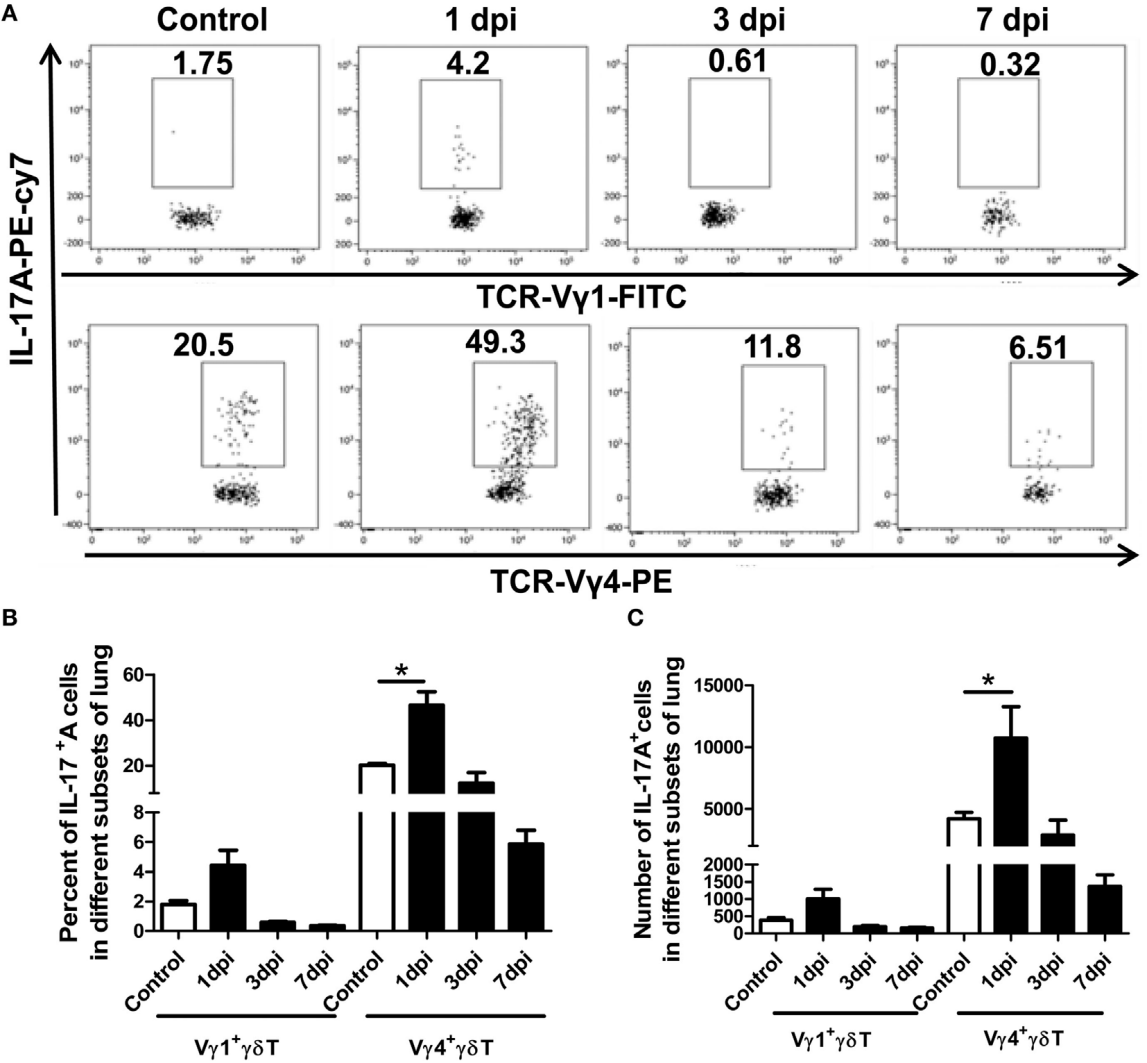
Expression of IL-17A in the Vγ1^+^γδT and Vγ4^+^γδT subsets. **(A)** Stained lung lymphocytes were first gated on CD3^+^γδTCR^+^ cells, and then the expression of IL-17A in the Vγ1^+^γδT subset (upper) and Vγ4^+^γδT subset (lower) in the control or infected lungs at the indicated dpi was analyzed. The numbers represent the proportion of IL-17A-positive cells among the different subsets at the indicated dpi. Histogram showing the changes in the proportion **(B)** and number **(C)** of IL-17A-positive cells among the Vγ1^+^γδT and Vγ4^+^γδT subsets in the control or infected lungs at different dpi. The results are representative of two independent analyses (*n* = 3 for each group). *p < 0.05.

IL-17A^+^γδT cells were significantly increased after infection, but the major subsets of IL-17A-secreting γδT cells in severe influenza virus infection were unclear. As shown in Figures 3A–C, the expression of IL-17A in Vγ1^+^γδT and Vγ4^+^γδT cells was detected using flow cytometry. The percentage of IL-17A-positive cells among the Vγ1^+^γδT and Vγ4^+^γδT subsets was significantly increased at 1 dpi and then attenuated at 3 and 7 dpi. Moreover, the proportion of IL-17A-positive cells among the Vγ4^+^γδT cells was far higher than that among the Vγ1^+^γδT cells. The proportion of IL-17A-positive cells among the Vγ1^+^γδT cells was 1.8, 4.4, 0.59, and 0.35%, but the proportion among the Vγ4^+^γδT cells was 20.2, 46.6, 12.4, and 5.9% in the uninfected control, 1, 3, and 7 dpi lungs, respectively (Figure 3B). The absolute number of IL-17A^+^Vγ1^+^γδT and IL-17A^+^Vγ4^+^γδT cells was significantly increased at 1 dpi and then declined at 3 and 7 dpi. Furthermore, the absolute number of IL-17A^+^Vγ4^+^γδT cells was far greater than the absolute number of IL-17A^+^Vγ1^+^γδT cells. The number of IL-17A^+^Vγ4^+^γδT cells (1.07 × 10^4^) was tenfold that of IL-17A^+^Vγ1^+^γδT cells (0.99 × 10^3^) at 1 dpi (Figure 3C). In contrast, in the spleen, only a slight increase in IL-17A-positive cells was observed among the γδT cells and the Vγ4^+^γδT subset, but not the Vγ1^+^γδT subset, at 1 dpi, and the percentage of IL-17A^+^γδT lymphocytes in the spleen was then gradually reduced to half the normal level at 3 and 7 dpi. This indicates the recruitment of γδT cells from the spleen to the lung after infection (Figure S3 in Supplementary Material; IL-17A^+^Vγ1^+^γδT subset flow chart and histogram not shown). These results demonstrated that Vγ4^+^γδT cells were the major source of IL-17A in the lungs at the early stage of severe influenza A (H1N1) pdm09 infection.

### IL-1β and IL-23 Were Each Sufficient to Induce IL-17A Production by γδT Cells

**Figure 4:**
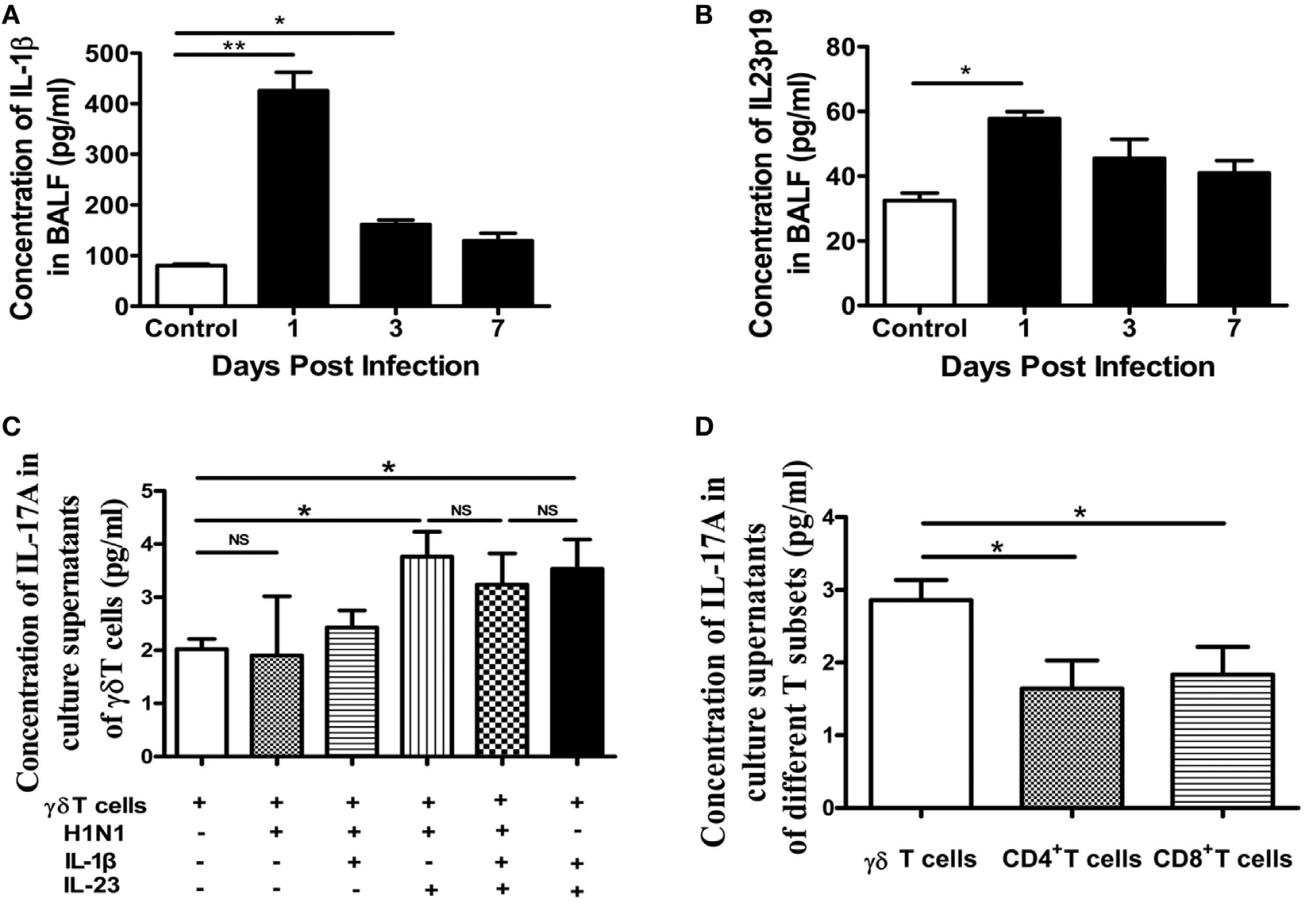
Expression of IL-17A by γδT cells was induced by IL-1β and/or IL-23. Concentrations of IL-1β **(A)** and IL-23 **(B)** in BALF of the control or infected mice at the indicated dpi were quantified using ELISA. **(C)** MACS-sorted lung-derived γδT cells from uninfected mice were stimulated with H1N1 virus (10^4^ TCID_50_/100 μl, 5 µl/well), IL-1β (10 ng/ml), and/or IL-23 (10 ng/ml) for 36 h, as indicated. Concentrations of IL-17A in the supernatants were quantified using ELISA. **(D)** MACS-sorted infected lung-derived γδT cells, CD4^+^T cells, or CD8^+^T cells at 1 dpi were cultured for 36 h, and concentrations of IL-17A in the supernatants were quantified using ELISA. *p < 0.05, **p < 0.01.

It is known that γδT cells can induce very fast, innate-like responses through TCR-dependent or -independent signaling, involving proinflammatory cytokines, the NKG2D receptor, or pattern-recognition receptors. It has been reported that the production of IL-17A by γδT cells appeared to be largely independent of TCR activation and IL-1β and IL-23 were sufficient to trigger abundant secretion of IL-17A by γδT cells (14). As shown in Figures 4A,B, the concentration of IL-1β and IL-23 in the BALF was significantly improved at the early stage of infection, which might promote the secretion of IL-17A by γδT cells.

We next explored whether the secretion of IL-17A by γδT cells was induced by the virus or cytokines. γδT cells, CD4^+^T cells, and CD8^+^T cells were isolated from lung using magnetic-activated cell sorting (MACS) and, then, stimulated with influenza A (H1N1) pdm09 virus, and IL-1β and/or IL-23. As shown in Figure 4C, γδT cells could only secret a small quantity of IL-17A when cultured with medium and the virus could not induce the expression IL-17A directly. However, IL-23 could induce a significant increase in IL-17A, IL-1β could induce a slight increase, and IL-23 and IL-1β had no synergistic effect. Thus, interestingly, the virus was not direct cause for inducing IL-17A production by γδT cells. Moreover, the virus, IL-23, or IL-1β could not induce the expression IL-17A by CD4^+^T cells and CD8^+^T cells (Figures S4A,B in Supplementary Material). To further confirm the dominantly producing of IL-17A by γδT cells, γδT cells, CD4^+^T cells, and CD8^+^T cells from infected lung at 1 dpi were isolated using MACS and, then, cultured to detect IL-17A in supernatants. As shown in Figure 4D, the concentration of IL-17A from the supernatants of cultured γδT cells was significantly higher than that of CD4^+^T cells or CD8^+^T cells. Moreover, the ability to secrete IL-17A of γδT cells was not further amplified by IL-23 or IL-1β (Figures S4C–E in Supplementary Material). These findings suggested that influenza A (H1N1) pdm09 virus might infect epithelial cells, macrophages, or dendritic cells and cause them to secret IL-1β and IL-23, which then promote IL-17A production by γδT cells in an antigen-independent manner.

### Pulmonary γδT Cells Could Also Secret a Certain Amount of IFN-γ

**Figure 5:**
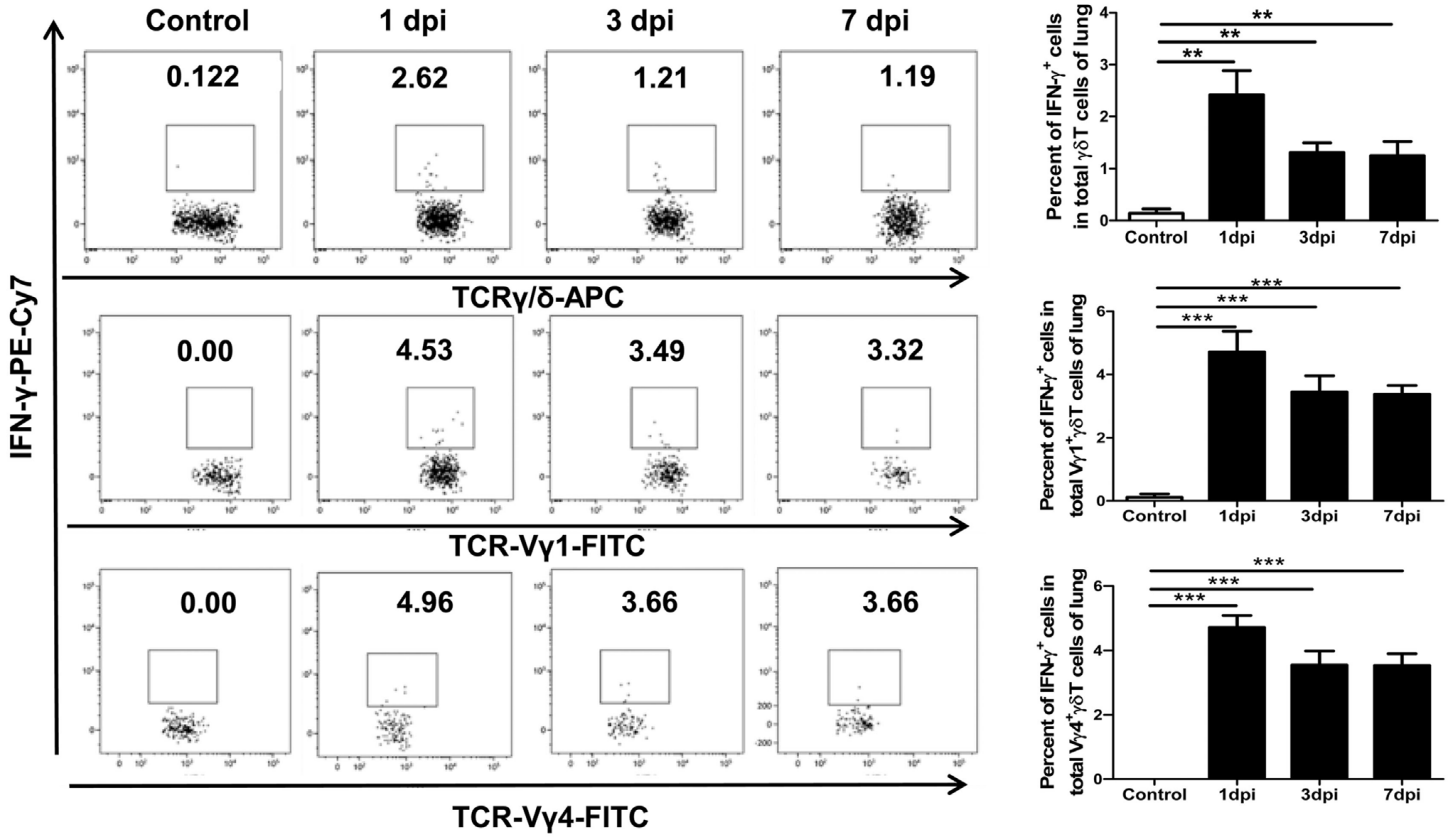
Expression of IFN-γ by γδT cells and Vγ1^+^γδT or Vγ4^+^γδT subset at different dpi in the lungs. The stained lung lymphocytes were first gated on CD3^+^T cells, and then, the expression of IFN-γ in γδT cells (upper) in the control or infected lungs at the indicated dpi were analyzed. The expression of IFN-γ in the Vγ1^+^γδT (middle) and Vγ4^+^γδT (lower) subsets was first gated on CD3^+^γδTCR^+^ cells. The numbers represent the proportion of IFN-γ-positive cells among the different subsets at the indicated dpi. Histogram showing the statistical analysis of the proportion of different subsets in the control or infected lungs at different dpi. The results are representative of two independent analyses (*n* = 3 for each group). **p < 0.01, ***p < 0.001.

Interferon-γ is an important early-phase antiviral effector cytokine secreted by activated γδT cells, which can act together with other immune effectors (particularly NK cells) for rapid and efficient virus control (25–27, 43). In the peripheral tissues, the functional orientation of γδT cells depends on the microorganism encountered, with IFN-γ production dominating antiviral responses (44). On the other hand, stimulation with IL-1β and IL-23 can also induce the co-production of IFN-γ by γδT17 cells (45). Flow cytometry was used to further analyze the expression of IFN-γ among γδT cells and their dominant subsets. As shown in Figure 5, the percentage of IFN-γ-positive cells among the γδT cells significantly increased from 0.14% in the uninfected lungs to 2.42, 1.31, and 1.25% in the infected lungs at 1, 3, and 7 dpi, respectively. The percentages of IFN-γ-positive cells among the Vγ1^+^γδT and Vγ4^+^γδT subsets all increased significantly after infection, with similar rates of increase; this was different from the IL-17A production, which was mainly derived from Vγ4^+^γδT cells. However, IFN-γ^+^γδT cells, as well as the IFN-γ^+^Vγ1^+^γδT and IFN-γ^+^Vγ4^+^γδT subsets, in the infected spleens gradually decreased compared with the levels in the uninfected mice (Figure S5 in Supplementary Material).

### Activated γδT Cells Induced Expression of NKG2D and FasL

**Figure 6:**
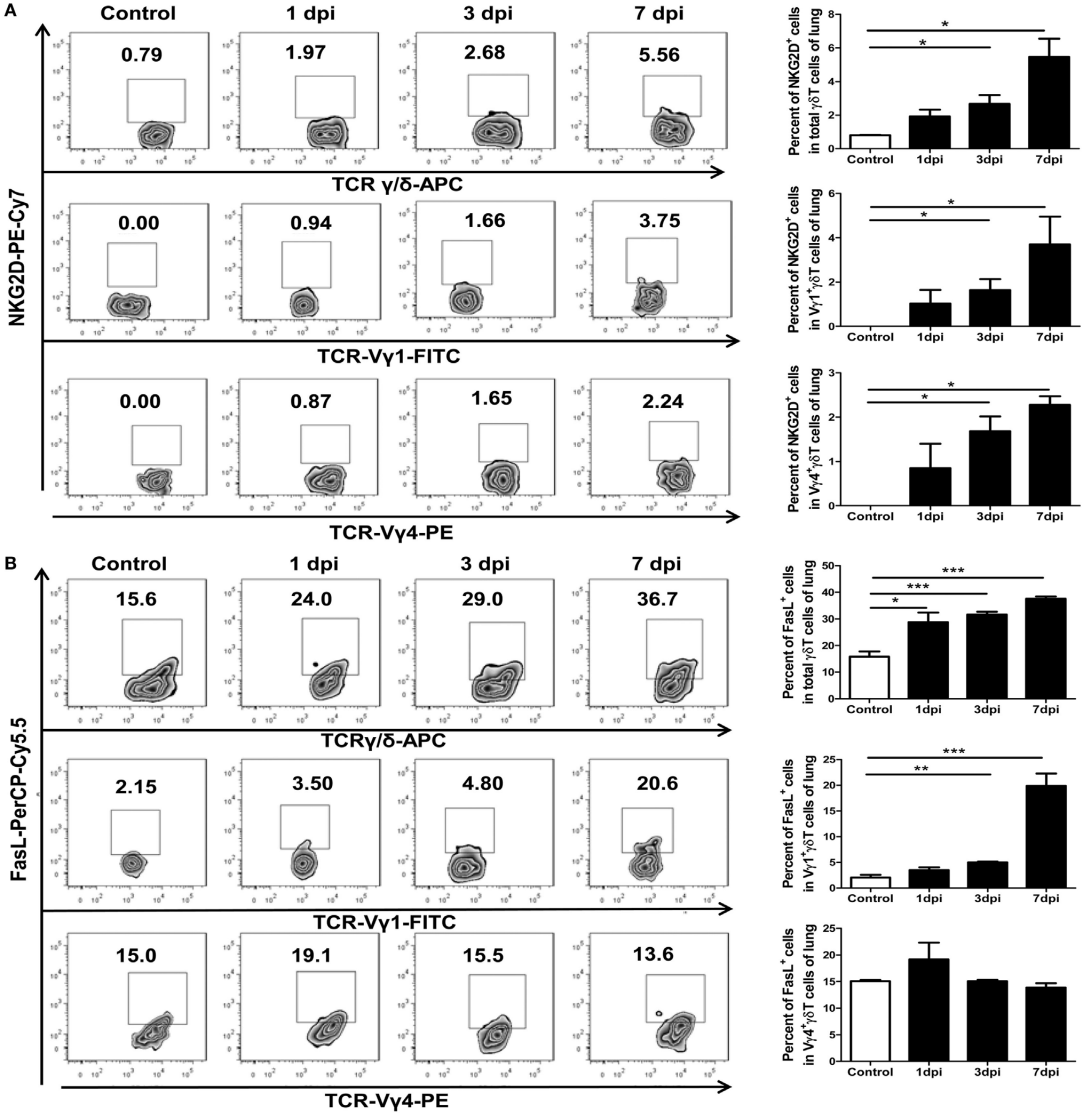
Influenza A (H1N1) pdm09 infection-induced expression of NKG2D and FasL in γδT cells and the Vγ1^+^γδT or Vγ4^+^γδT subset in the lungs at different dpi. Expression of NKG2D **(A)** and FasL **(B)** by γδT cells (upper, gated on CD3^+^T cells) and Vγ1^+^γδT (middle, gated on CD3^+^γδTCR^+^ cells) or Vγ4^+^γδT (lower, gated on CD3^+^γδTCR^+^ cells) subsets was measured using flow cytometry. The numbers represent the proportion of NKG2D- or FasL-positive cells among the different subsets at the indicated dpi. Histogram showing the statistical analysis of the proportion of NKG2D^+^ or FasL^+^ cells in the different subsets in the control or infected lungs at different dpi. The results are representative of two independent analyses (*n* = 3 for each group). *p < 0.05, **p < 0.01, ***p < 0.001.

It has been reported that human Vγ9Vδ2 T cells can be directly activated by NKG2D (46). Activated γδT cells can lyse and eliminate virus-infected cells by enhancing their cytotoxicity effector functions (44). It has also been reported that human Vγ9Vδ2 T cells can recognize and efficiently kill human or avian influenza virus-infected monocyte-derived macrophages. The cytotoxicity of Vγ9Vδ2 T cells is dependent on NKG2D activation and is mediated by the Fas–FasL and perforin–granzyme B pathways (27–30). To determine the phenotypes of the γδT cells and their subsets in the mice lungs, we examined the cell surface markers NKG2D and FasL in γδT cells from both control and infected mice lungs using flow cytometry. The percentage of NKG2D-positive cells among lung γδT cells, and Vγ1^+^γδT and Vγ4^+^γδT subsets, gradually increased, and there were significant differences at 3 and 7 dpi compared to the levels in the uninfected lungs (Figure 6A). Similar results were found in the infected γδT cells, and Vγ1^+^γδT and Vγ4^+^γδT subsets, in the spleens, but the positive rates were far lower than in the lungs (Figure S6A in Supplementary Material).

As the infection process, the percentage of FasL-positive cells among lung γδT cells and Vγ1^+^γδT subsets gradually increased but not among the Vγ4^+^γδT subset (Figure 6B). The percentage of FasL-positive cells among γδT cells was significantly raised from 15.8% in the uninfected lungs to 28.7, 31.6, and 37.6% in the infected lungs at 1, 3, and 7 dpi, respectively (Figure 6B). The FasL-positive cells in the infected γδT cells, Vγ1^+^γδT and Vγ4^+^γδT subsets, in the spleens were only significant increased at 1 dpi, and the positive rates were far lower than that in the lungs (Figure S6B in Supplementary Material).

### Depletion of γδT Cells or the Vγ4^+^γδT Subset But Not the Vγ1^+^γδT Subset Alleviated Lung Injury and Increased the Survival Rate

**Figure 7:**
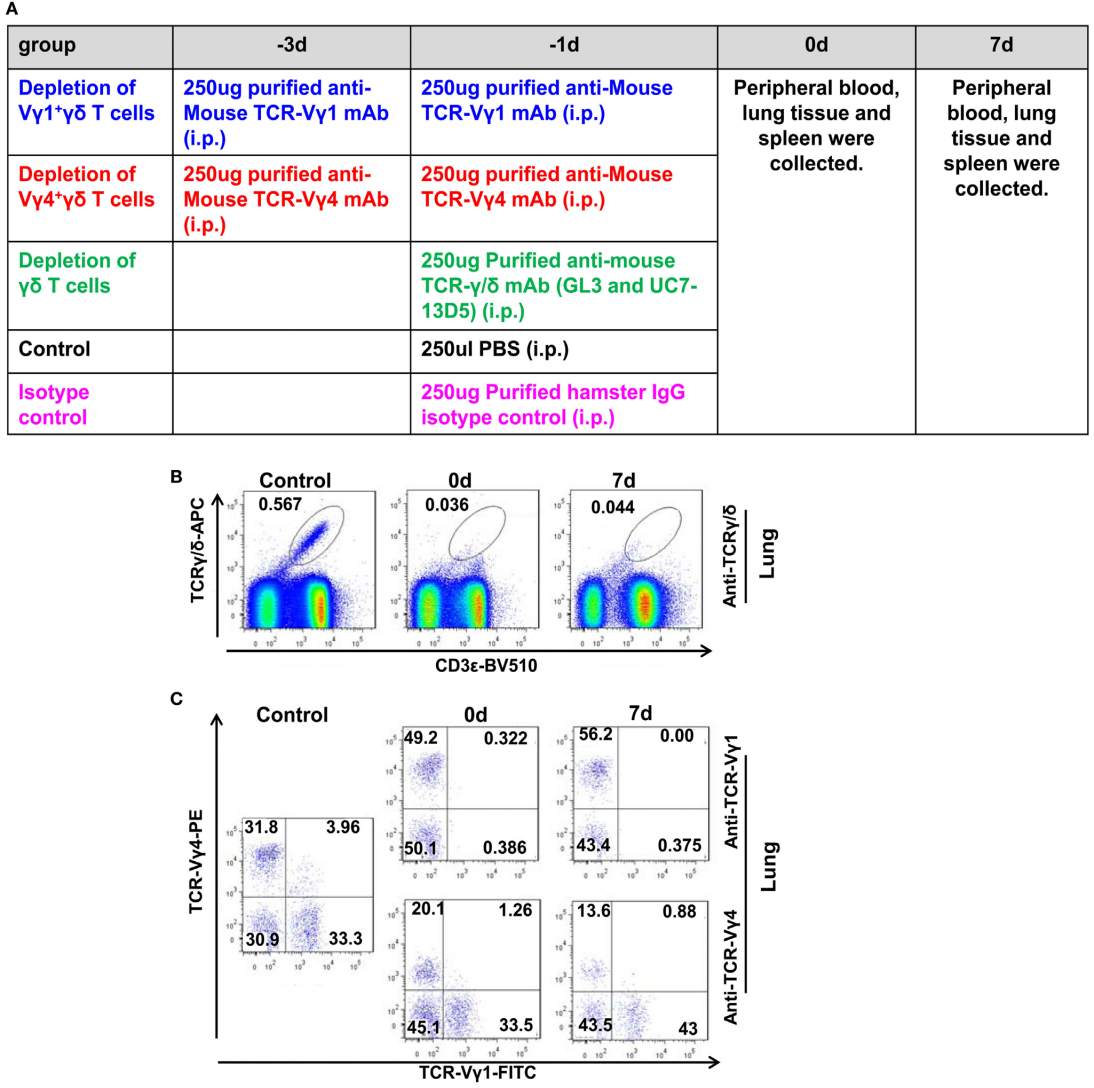
Depletion of γδT cells and the Vγ1^+^γδT or Vγ4^+^γδT subsets. **(A)** Schematic diagram of the methods used to deplete the indicated cells, based on the detailed description in Section “Materials and Methods.” Depletion of lung γδT cells [**(B)** gated on lung lymphocytes] and the Vγ1^+^γδT or Vγ4^+^γδT subsets [**(C)** gated on lung CD3^+^γδTCR^+^ lymphocytes] was detected using flow cytometry at 0 and 7 dpi. The numbers represent the proportion of different subsets in the lungs after depletion. The results are representative of two independent analyses (*n* = 3 for each group).

After we confirmed that γδT cells and the Vγ4^+^γδT subset were the major source of the elevated IL-17A that mediated lung immunopathological injury in severe infection, we further analyzed whether depletion of γδT cells or the Vγ4^+^γδT subset could alleviate lung injury. The neutralizing antibodies against TCRγ/δ, TCR Vγ4, TCR Vγ1 or isotype control were i.p. injected, as shown in Figure 7A. After anti-TCRγ/δ mAb treatment at 1 day before infection, 92.9% of γδT cells in the lungs were depleted on the day of infection (the percentage of γδT cells in the lungs was decreased from 0.57% in the PBS-treated control group to 0.04%), and the depletion lasted until 7 dpi (Figure 7B). For Vγ1^+^γδT and Vγ4^+^γδT subset depletion, anti-TCR Vγ1 or anti-TCR Vγ4 mAbs, respectively, were injected twice on 3 and 1 days before infection. The results showed that almost all the lung Vγ1^+^γδT subset was depleted on the day of infection, and this could also last until 7 dpi (Figure 7C). In addition, 51.88 and 62.42% of the lung Vγ4^+^γδT subset were depleted on the day of infection and 7 dpi, respectively (Figure 7C). Meanwhile, almost all the Vγ1^+^γδT or Vγ4^+^γδT subsets were depleted in the blood and spleen on the day of infection and 7 dpi (Figure S7 in Supplementary Material).

**Figure 8:**
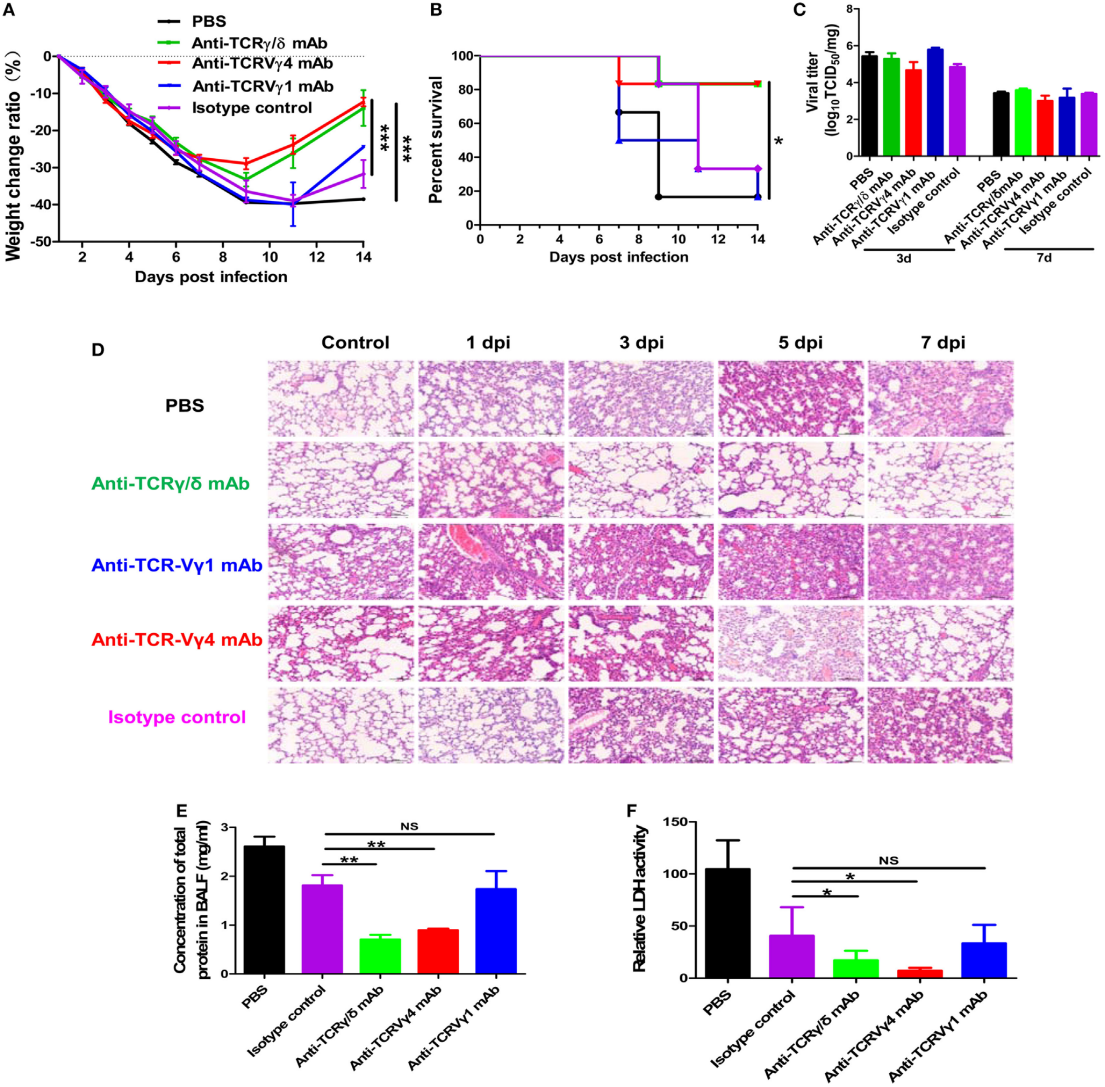
Depletion of γδT cells or the Vγ4^+^γδT subset reduced inflammatory damage and improved the survival rate. Weight change **(A)** and survival rate **(B)** of the PBS or isotype control group, γδT cell depletion group, Vγ1^+^γδT cell depletion group, and Vγ4^+^γδT cell depletion group after intranasal inoculation with influenza A (H1N1) pdm09 virus (*n* = 6 for each group). **(C)** Viral titer in the lungs of different cell depletion groups determined at 3 and 7 dpi. **(D)** Representative hematoxylin and eosin staining of lung sections from the control group and the γδT, Vγ1^+^γδT, and Vγ4^+^γδT cell depletion groups at the indicated dpi. Original magnification: ×200. Total protein concentration **(E)** and relative LDH activity **(F)** in BALF from the control group and different cell depletion groups at 3 dpi. *p < 0.05, **p < 0.01, ***p < 0.001.

Depletion of γδT cells or the Vγ4^+^γδT subset but not the Vγ1^+^γδT subset significantly reduced the severe influenza A (H1N1) pdm09 infection-induced weight loss (Figure 8A) and also significantly improved the survival rate (Figure 8B). The survival rate was only 16.7% at 14 dpi [with 10^2^ TCID_50_ influenza A (H1N1) pdm09 virus], but it was significantly improved (to 83.3%) at 14 dpi after γδT cell depletion. Surprisingly, depletion of the Vγ4^+^γδT subset led to a similar survival rate to that after depletion of the γδT cells, but depletion of the Vγ1^+^γδT subset did not change the survival rate (Figure 8B).

Further analysis showed that the improved survival rate was not related to the viral load but to reduced ALI. Compared to the PBS or isotype control group, the viral titers at 3 and 7 dpi were similar in the γδT, Vγ1^+^γδT, and Vγ4^+^γδT cell depletion groups (Figure 8C). Histopathological analysis of the lungs of the PBS or isotype antibody-treated control mice showed gradually increased histopathological injury and a large amount of infiltrating inflammatory cells. Depletion of γδT cells significantly ameliorated the lung immunopathological injury and attenuated the inflammatory cell infiltration (Figure 8D). Consistent with the survival rate findings, depletion of the Vγ4^+^γδT subset but not the Vγ1^+^γδT subset also ameliorated lung injury and attenuated inflammatory cell infiltration (Figure 8D). In addition, the concentration of total protein in the BALF also decreased from 2.61 mg/ml in the control group to 0.71 and 0.89 mg/ml in the γδT and Vγ4^+^γδT cell depletion groups at 3 dpi, respectively (Figure 8E). Moreover, the activity of LDH in the BALF was decreased from 104.56 U/l in the control group to 17.18 and 7.06 U/l in the γδT and Vγ4^+^γδT cell depletion groups, respectively (Figure 8F).

### Depletion of γδT Cells or Vγ4^+^γδT Subset Significantly Decreased the Concentration of IL-17A at the Early Stage of Infection

**Figure 9:**
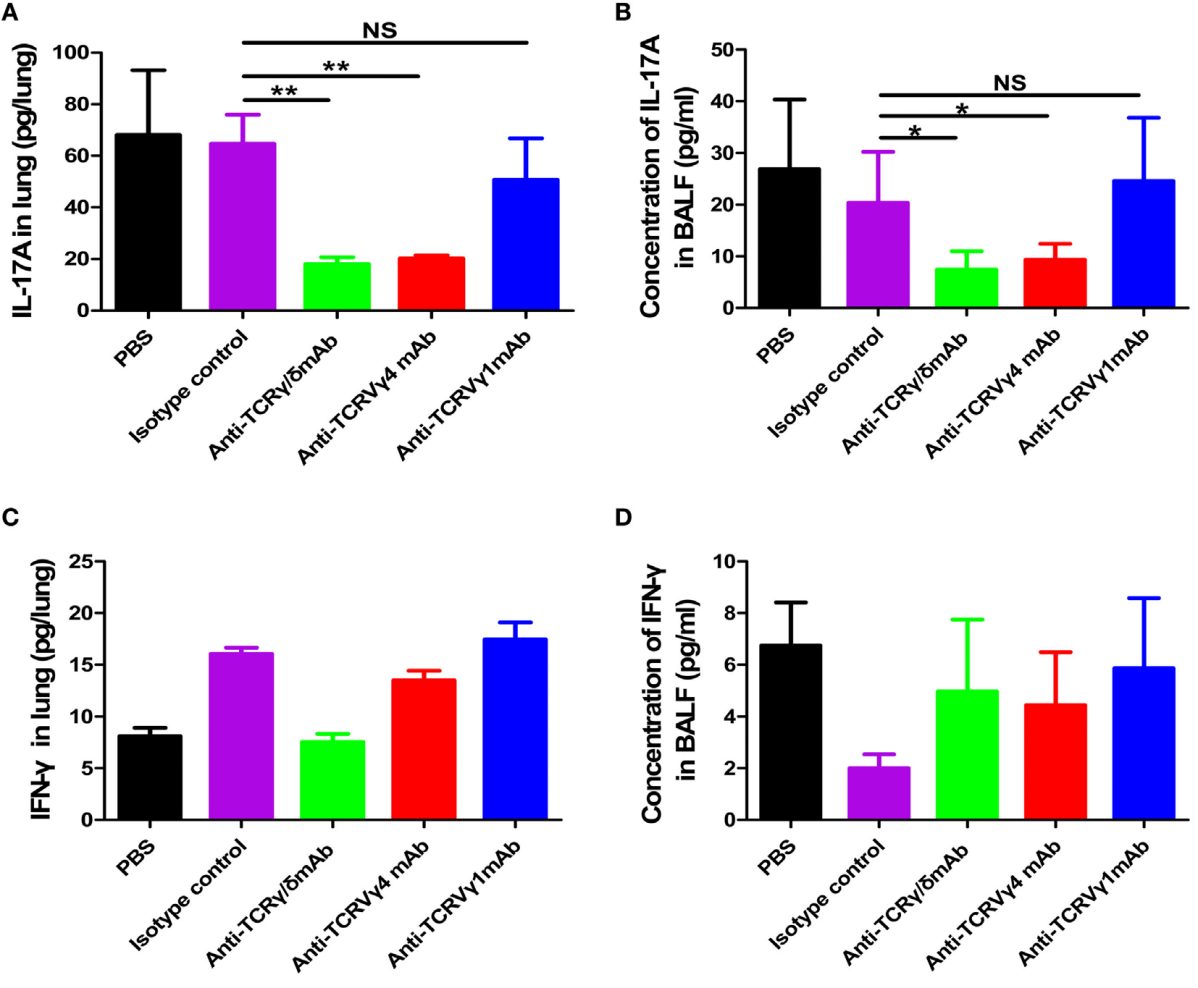
Reduced IL-17A secretion in γδT cells and Vγ4^+^γδT subset depletion groups. The amount of IL-17A **(A)** or IFN-γ **(C)** in lung and the concentration of IL-17A **(B)** or IFN-γ **(D)** in BALF from the PBS control group, isotype antibody control group, γδT cell depletion group, Vγ1^+^γδT cell depletion group, and Vγ4^+^γδT cell depletion group were quantified using ELISA at 1 dpi. **p < 0.01. NS, not significant.

We next detected whether the alleviated lung injury in the γδT or Vγ4^+^γδT cell depletion groups was related to changes in cytokines. As shown in Figures 9A,B, depletion of γδT cells or the Vγ4^+^γδT subset significantly decreased the amount of IL-17A in lung and the concentration of IL-17A in BALF compared to PBS or isotype antibody control group. The amount of IL-17A in lung of γδT cells depletion group and Vγ4^+^γδT subset depletion group were decreased to 27.91 and 31.21% of that in isotype antibody control group, respectively. Meanwhile, the concentrations of IL-17A in BALF of γδT cells depletion group and Vγ4^+^γδT subset depletion group were also reduced to 30.5 and 36%, respectively, when compared to isotype antibody control group. However, depletion of Vγ1^+^γδT subset showed similar level IL-17A in lung and BALF with PBS or isotype antibody control group. Since pulmonary γδT cells could also secret a small amount of IFN-γ, the levels of IFN-γ in lung and BALF of different depletion groups were also detected. As shown in Figures 9C,D, depletion of γδT cells, as well as Vγ1^+^γδT and Vγ4^+^γδT subsets, had no obvious changes when compared with PBS and isotype control groups. As intraperitoneal injection of depletion antibodies depletes systemic γδT lymphocytes, the IL-17A and IFN-γ in serum were also detected. As shown in Figure S8A in Supplementary Material, depletion of γδT cells or the Vγ4^+^γδT subset significantly decreased the serum concentration of IL-17A to 14.3 and 23.5% of the concentrations in the PBS-treated control group at 3 dpi. Puzzlingly, depletion of the Vγ1^+^γδT subset also decreased the concentration of IL-17A, which might be due to systemic depletion. Meanwhile, the concentration of IFN-γ also showed a slightly lower after depletion of γδT, Vγ1^+^γδT, and Vγ4^+^γδT cells at 2 dpi compared to isotype control group, but there was a sharp rise at 7 dpi, which might have been due to the differentiation of Th1 cells (Figure S8B in Supplementary Material).

## Discussion

Influenza virus infections are a leading cause of morbidity and mortality worldwide. Infected young- or middle-aged people tend to have more severe edema of the trachea, diffuse alveolar injury, and inflammatory cell infiltration, emphasizing the importance of understanding the pathogenesis of influenza-induced ALI (4, 5, 47). Aggressive inflammation is thought to cause most of the damage in the lungs during infection. Cytokines, especially IL-17A, play an important role in lung immunopathological injury at the early stage of influenza virus infection (12, 48). Searching for the major IL-17A secretory cells and clarifying their mechanisms will help us to better understand the pathogenesis of influenza virus-induced severe ALI.

In this study, we found that γδT cells (including the Vγ4^+^γδT subset) were rapidly recruited to the lungs (Figures 1E,G) and were the major source of lung IL-17A (Figures 2 and 3), which might mediate the development ALI and ARDS at the early stage during severe influenza A (H1N1) pdm09 virus infection in mice. It was also proved that the secretion of IL-17A by activated γδT cells was directly induced by IL-1β and/or IL-23 cytokines, rather than the virus (Figure 4). In addition to secreting IL-17, γδT cells increased the expression of the activation-associated molecule NKG2D and the cytotoxicity-associated molecule FasL (Figure 6). Importantly, depletion of γδT cells or the Vγ4^+^γδT subset prior to infection significantly reduced the mortality and alleviated influenza A virus-induced ALI in mice (Figure 8).

The lung is an important organ of the respiratory system, and a large number of pathogens can be inhaled when breathing in. The lung is rich in innate immune cells, which can rapidly and effectively eliminate pathogens to maintain the immune balance. Unlike αβT cells and B cells, γδT cells are mainly located in non-lymphoid tissue, such as the epithelium of mucosal tissue. The proportions of γδT cells in the lungs, throat, nose, mouth, and small intestines are much higher than the proportions in the lymph nodes and spleen (49). This specific distribution suggests that γδT cells are the first line of host defense against infection. γδT cells have displayed potential antiviral activities against many viruses, such as human cytomegalovirus (CMV) (50), hepatitis B virus (HBV) (51), and simian immunodeficiency virus (SIV) (52). γδT cell-based therapy has great potential for the treatment of infectious diseases (53).

Our results showed that the accumulation of γδT cells and the Vγ4^+^γδT subset in the lungs was accompanied by reductions in the spleens. And significant increases in the antiviral-associated molecules FasL and NKG2D, as well as IFN-γ, were found in the lungs after infection. This suggested that the migration of γδT cells from the spleen to the lungs might be a reason for the increased level of γδT cells. Chemokine receptors, for example, C-C chemokine receptor 5 (CCR5), can be expressed on γδT cells (54, 55). Significantly increased levels of regulated on activation, normal T cell expressed and secreted (RANTES), macrophage inflammatory protein-1α (MIP-1α), and MIP-1β were detected in the BALF of the mice severely infected with influenza A (H1N1) pdm09 virus (data not shown), which could mediate γδT cell diapedesis into inflammatory sites. Peripheral γδT cells that appear to be “resting yet activated” might be another reason for the increase in γδT cells and the Vγ4^+^γδT subset in the lungs (56). However, overactive γδT cells might have immunopathological effects during severe infection due to the secretion proinflammatory cytokines or the expression of activated receptors.

Interleukin-17 is a very important inflammatory cytokine, and it plays both pathological and protective roles in inflammatory diseases (57). CD4^+^T cells are initially believed to be the primary source of IL-17, but subsequent research shows that γδT cells are another more potent source of IL-17 during the early stage of immune responses (58). We found that γδT17 cells but not Th17 and Tc17 cells were the principal source of IL-17 at the early phase of severe influenza A (H1N1) pdm09 virus infection. This finding is similar to the findings of Hamada et al. from a study on the livers of mice infected with *L. monocytogenes*, and they also found that the proportion of IL-17A^+^γδT cells (~20–34%) was significantly higher than the proportion of IL-17A^+^αβT cells (<1%) (23). Given the major contributions of γδ17 T cells to early stage of immune responses, the developmental and functional relationships between them and other innate sources of IL-17, such as “NKT17” NKT cells or type 3 innate lymphoid (ILC3) cells, should be investigated further.

However, the percentage and number of γδT17 were then gradually reduced. With the initiation of the adaptive immune response, naive T cell that differentiate into Th17 and Tc17 cells might be a source of IL-17 during the late phase of infection. The reason for this phenomenon may be that IL-17A produced by γδT17 cells in the lungs promotes IL-17 expression by Th cells (14). γδT17 cells are typically found in lungs and secondary lymphoid organs, and Vγ4^+^γδT cells are one of the major subsets that produce IL-17 in various experimental models (41). Our study also found that the Vγ4^+^γδT subset but not the Vγ1^+^γδT subset was the main source of IL-17 in the lungs of mice severely infected with influenza A (H1N1) pdm09. Similarly, the Vγ4^+^γδT and Vγ6^+^γδT subsets were also previously found to produce a large amount of IL-17 in a model *of M. tuberculosis* infection (31). Another IL-17 family member, IL-17C, is a critical factor that potentiates inflammatory responses and causes host injury during fungal infection (59), and further investigation is needed to explore its role during influenza virus infection.

A recent report demonstrated that influenza virus infection could induce increased IL-17A expression in mice and then caused immune injury not only in respiratory tissues, but also in intestinal mucosal tissues (60). However, the IL-17 principally produced by conventional Th17 cells was responsible for gut-associated damage (60). We believe that there are three main reasons to explain the differences between the previous study and the current study: (1) different immuno-microenvironments in different mouse strain models; (2) the body weight loss as the infection progressed was different, which might have resulted from different virus strains and different infective doses; and (3) our results focused on the innate immune response of ALI at 1 dpi, while the previous study focused on the adaptive immune response involving Th17 cell polarization at 7 dpi.

γδT cells can be activated in a TCR-dependent or cytokine-dependent (TCR-independent) manner. Martin et al. argue that γδT cells can directly recognize pathogen-associated patterns and trigger the initiation of IL-17 production and neutrophil chemotaxis and IL-23 secreted by antigen-presenting cells can promote the proliferation of γδT17 cells (61). IL-23 combined with IL-1β promotes the expression of IL-17 by γδT cells in the absence of additional signals in experimental autoimmune encephalomyelitis (14). In a tumor microenvironment, γδT cells in the lungs can also be activated and secrete IL-17, in a γδTCR-independent and IL-1β- and IL-23-dependent manner (62). To further explore the possible mechanisms of γδT cell IL-17 production after influenza virus infection, purified lung-derived γδT cells were cultured *in vitro* with either IL-1β, IL-23, IL-1β, and IL-23 together or influenza virus. Our results showed that IL-23 and IL-1β were each sufficient to induce IL-17A production by γδT cells but influenza virus was not. The concentrations of IL-1β and IL-23 were significantly increased at the early stage of infection (Figures 4A,B), suggesting that other innate immune cells infected with influenza virus (alveolar macrophages, dendritic cells, alveolar epithelial cells, etc.) might secret proinflammatory cytokines to promote IL-17 secretion by γδT cells.

Interferon-γ is another important antiviral cytokine that can be secreted by γδT cells during influenza virus infection. The proportion of IFN-γ^+^γδT cells in the lungs was significantly increased after infection, and the Vγ1^+^γδT and Vγ4^+^γδT subsets had a similar ability to secrete IFN-γ. This situation was different from IL-17A production, which was predominantly derived from Vγ4^+^γδT cells. He et al. reported that CD44-rich Vγ4^+^γδT cells secreted more IFN-γ than Vγ1^+^γδT cells, partly due to the high expression of eomesodermin (63). In contrast, Vγ1^+^γδT cells were the major γδT subset that produced IFN-γ in response to *L. monocytogenes* infection (64). These conflicting results are likely due to different disease models and treatment methods (65).

Cell activation and apoptosis-related molecules were also expressed by γδT cells, such as NKG2D and FasL. Influenza virus infection can induce the expression of the NKG2D ligand in the lungs (data not shown). It has been reported that NKG2D can directly activate Vγ9Vδ2 T cells and trigger their release of cytolytic granules due to the recognition of NKG2D (46). NKG2D is also involved in the lysis of tumor cells by γδT cell-mediated cytotoxicity in tumor models (66, 67) The Fas–FasL pathway is also involved in the destruction of *L. monocytogenes*-infected macrophages by murine γδT cells *in vivo* (68). The cytotoxicity of Vγ9Vδ2 T cells against influenza virus-infected monocyte-derived macrophages is found to be dependent on NKG2D activation and mediated by the Fas–FasL and perforin–granzyme B pathways (30). In our study, the proportion of NKG2D^+^γδT cells, as well as the proportions of the NKG2D^+^Vγ1^+^γδT and NKG2D^+^Vγ4^+^γδT subsets, gradually increased during the influenza virus infection, which might underlie the activation of the γδT cells and the cytotoxicity of virus-infected cells. The expression of FasL on γδT and Vγ1^+^γδT cells was also increased after influenza virus infection, which might have induced apoptosis of the infected target cells. These results demonstrate that γδT cells might play cytotoxic roles due to increased expression of NKG2D and FasL during the late stage of infection.

Based on our previous reports on the immunopathological roles of IL-17A and our current results that IL-17A originates from γδT cells during the early stage of infection, the immunopathological roles of γδT cells, as well as the Vγ1^+^γδT and Vγ4^+^γδT subsets, were further explored in the early phase of severe influenza virus infection. Anti-TCR γδ mAbs (clone no. GL3 and UC7-13D5) and anti-TCR Vγ1 mAbs (clone no. 2.11) depleted almost all the γδT cells and the Vγ1^+^γδT subset, respectively, in the lungs, spleen, and peripheral blood. Anti-TCR Vγ4 mAbs (clone no. UC3-10A6) depleted almost all the Vγ4^+^γδT subset in the spleen and peripheral blood but not in the lungs. The undepleted Vγ4^+^γδT cells might been tissue-resident lymphocytes, which suggest that they have the ability to rapidly produce effector cytokines and cytolytic molecules upon activation during the early stage of the influenza virus infection. Additional effective depletion antibodies against TCR Vγ4 need to be explored further.

In our study, depletion of γδT cells or the Vγ4^+^γδT subset before viral infection significantly improved the survival rate and reduced the inflammatory lung tissue damage but did not reduce the viral titers. In parallel, compared to that in the control mice, the serum level of IL-17A was significantly lower in the γδT, Vγ1^+^γδT, and Vγ4^+^γδT cell depletion groups. These results demonstrate that γδT cells and the Vγ4^+^γδT subset promote immunopathological injury by secreting IL-17 rather than by directly clearing the virus during the early stage of severe infection with influenza A virus. However, when γδT cells were depleted with mAbs specific for TCRγδ, different results might be obtained depending on the timing of administration (69, 70).

In conclusion, our findings demonstrated that γδT cells and the Vγ4^+^γδT subset were recruited to the lungs and exacerbated ALI by secreting IL-17A during the early phase of severe influenza A virus infection. This mechanism provides a promising new target for the prevention and treatment of ALI induced by severe influenza A (H1N1) pdm09 infection.

## Ethics Statement

This study was carried out in accordance with the recommendations of Chinese National Guidelines for the Care of Laboratory Animals and the Institutional Animal Care and Use Committee of the Institute of Laboratory Animal Science, Peking Union Medical College. The protocol was approved by the Institutional Animal Care and Use Committee of the Institute of Laboratory Animal Science, Peking Union Medical College (ILAS-PC-2015-016).

## Author Contributions

XZ, BC, YA and MW conceived and designed. CX, MW, LB, FL, HL, ML and QL acquired, analyzed and interpreted the information. CX, XZ, BC, HL and LB wrote, reviewed, and/or revised the manuscript. XZ, BC and CX proofread and formatted.

## Conflict of Interest Statement

The authors declare that the research was conducted in the absence of any commercial or financial relationships that could be construed as a potential conflict of interest.

## Abbreviations

TCID50, 50% tissue culture infective dose; dpi, days post-infection; IL, interleukin; IFN, interferon.
