# Supplementary material for: Vγ4+γδT Cells Aggravate Severe H1N1 Influenza Virus Infection-Induced Acute Pulmonary Immunopathological Injury via Secreting Interleukin-17A
Source: Front Immunol. 2017 Aug 31;8:1054. doi: 10.3389/fimmu.2017.01054 (PMC5583159; doi:10.3389/fimmu.2017.01054)
Supplement: Supplementary file 1 [file Data_Sheet_1.DOCX]

Supplementary Materials

**Vγ4^+^γδT cells aggravated** **severe H1N1 influenza virus infection induced acute pulmonary immunopathological injury *via* secreting IL-17A**

**Chunxue Xue, Mingjie Wen, Linlin Bao, Hui Li, Fengdi Li, Meng Liu, Qi Lv, Yunqing An, Xulong Zhang^*^, Bin Cao^*^**

*** Correspondence: Bin Cao M.D., E-mail:** [**caobin_ben@vip.163.com**](mailto:caobin_ben@vip.163.com) **or Xulong Zhang M.D., E-mail:** [**zhxlwl@ccmu.edu.cn**](javascript:;)

**Supplementary figures and figure legends**

**
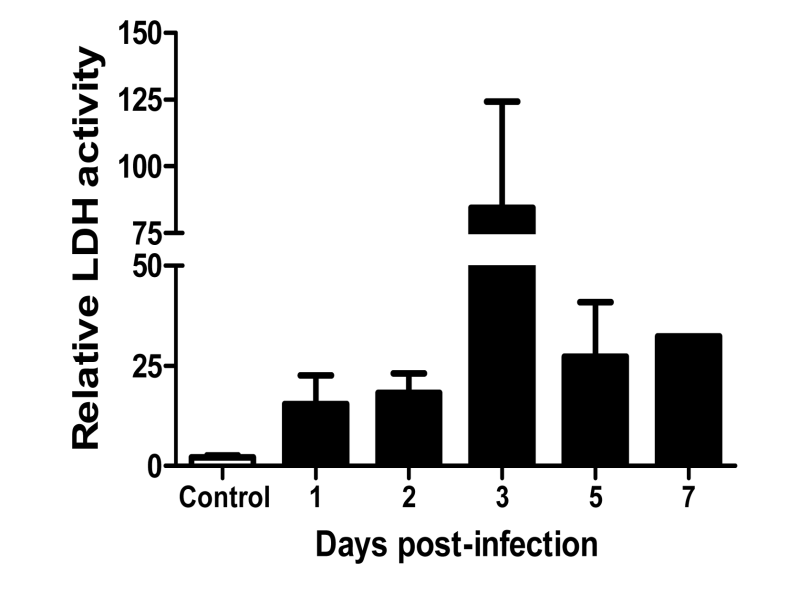
**

**Supplementary figure 1.** Severe influenza A (H1N1) pdm09 virus infection induced lung damage. The changes of relative lactate dehydrogenase (LDH) activity in BALF of infected mice were assayed at indicated dpi.


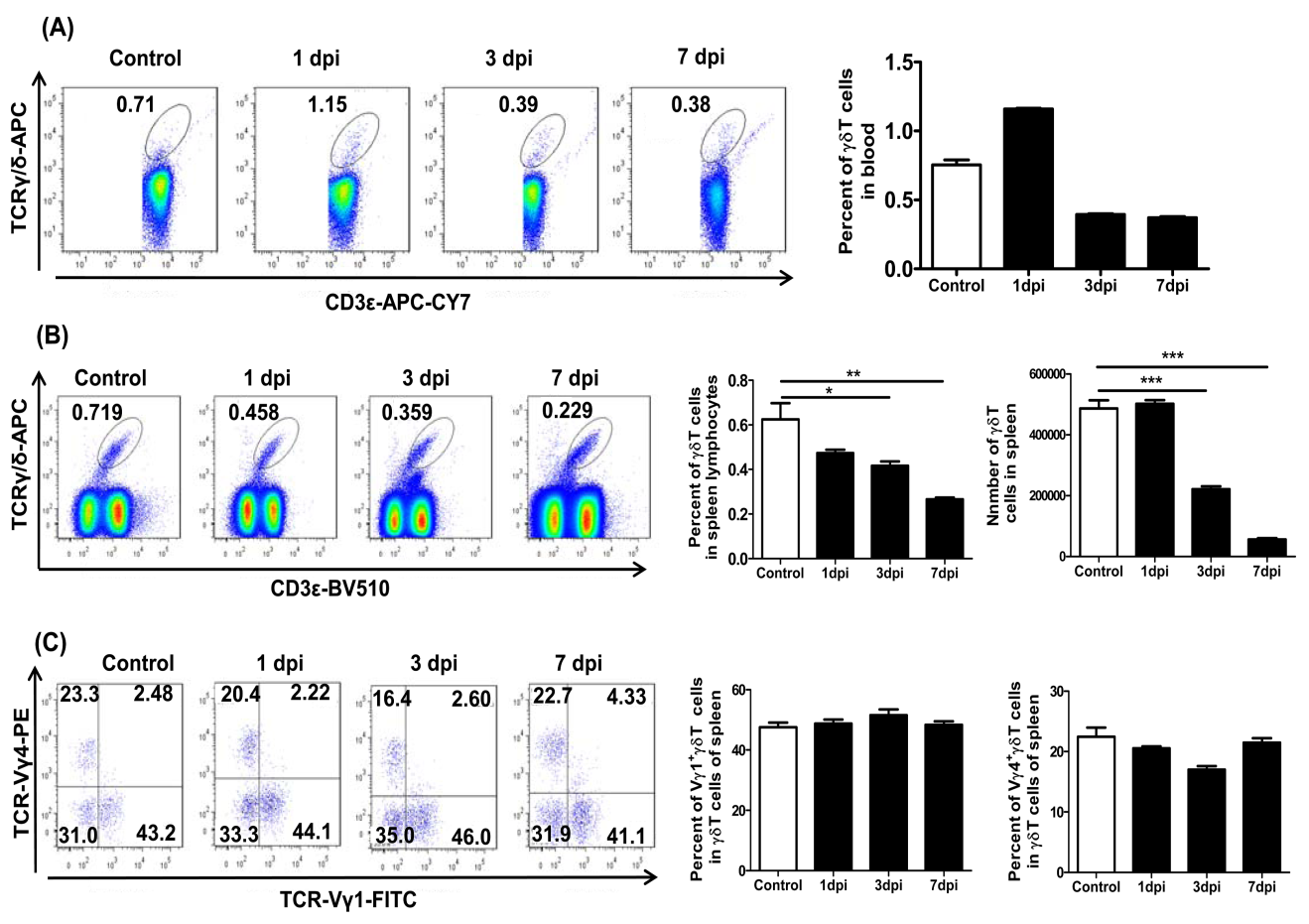


**Supplementary figure 2.** The changes of γδT cells, Vγ1^+^γδT and Vγ4^+^γδT subsets in blood and spleens after infection were detected by flow cytometry at indicated dpi. (A) The percentage of CD3^+^TCRγδ^+^ T cells in blood of control or 1, 3 and 7 dpi mice were detected by flow cytometry. (B) The percentage and number of CD3^+^TCRγδ^+^ T cells in the spleens of control or 1, 3 and 7 dpi mice were detected by flow cytometry. (C) The percent of Vγ1^+^γδT and Vγ4^+^γδT subset cells in the spleens of infected mice at indicated dpi. The stained spleen lymphocytes were first gated on CD3^+^TCRγδ^+^ cells, and then analyzed the subsets of Vγ1^+^γδT and Vγ4^+^γδT cells. The results were representative of two independent studies (n=3). The number signed in flow chart represented the ratio of indicated subsets. The statistical analysis was showed as **P*<0.05, ***P*<0.01, ****P*<0.001.


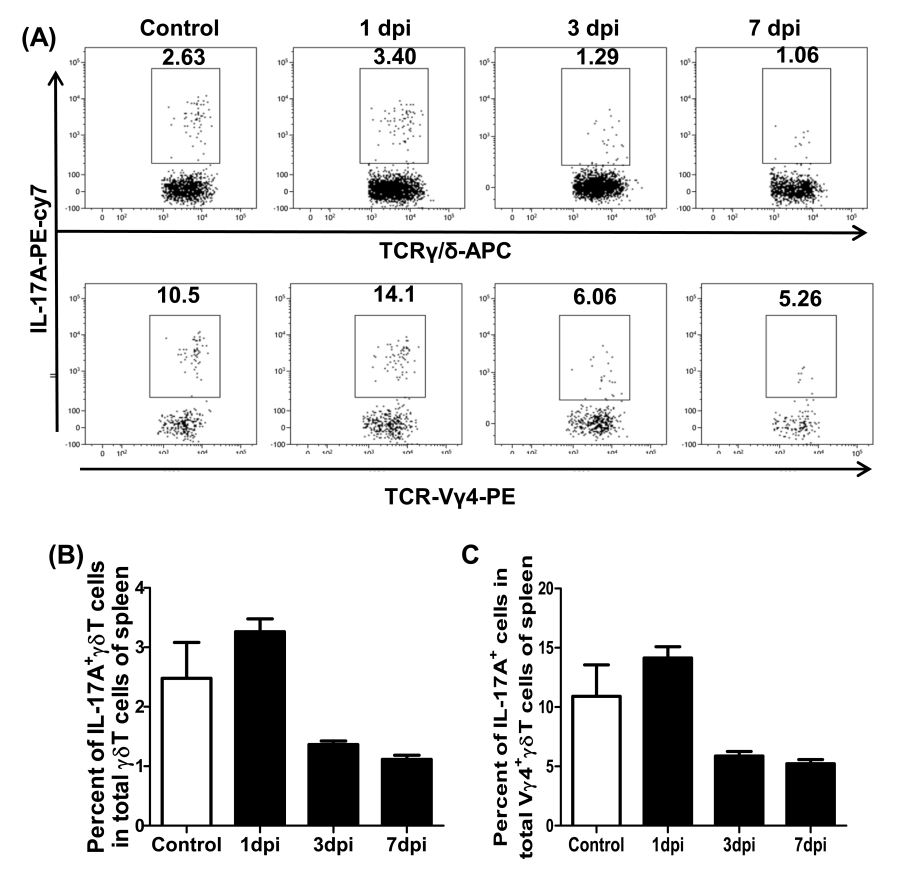


**Supplementary figure 3.** The expression of IL-17A in spleen γδT cells and Vγ4^+^γδT subsets. (A) The stained spleen lymphocytes were first gated on CD3^+^ cells, and then analyzed the expression of IL-17A in γδT cells (upper). The stained spleen lymphocytes were first gated on CD3^+^γδTCR^+^ cells, and then analyzed the expression of IL-17A in Vγ4^+^γδT subsets (below) in control or infected mice at indicated dpi. The number represented the ratio of IL-17A positive cells in different subsets at indicated dpi. Histogram showed the percent of IL-17A positive cell in γδT cells (B) and Vγ4^+^γδT subsets (C) in control or infected lung at different dpi.


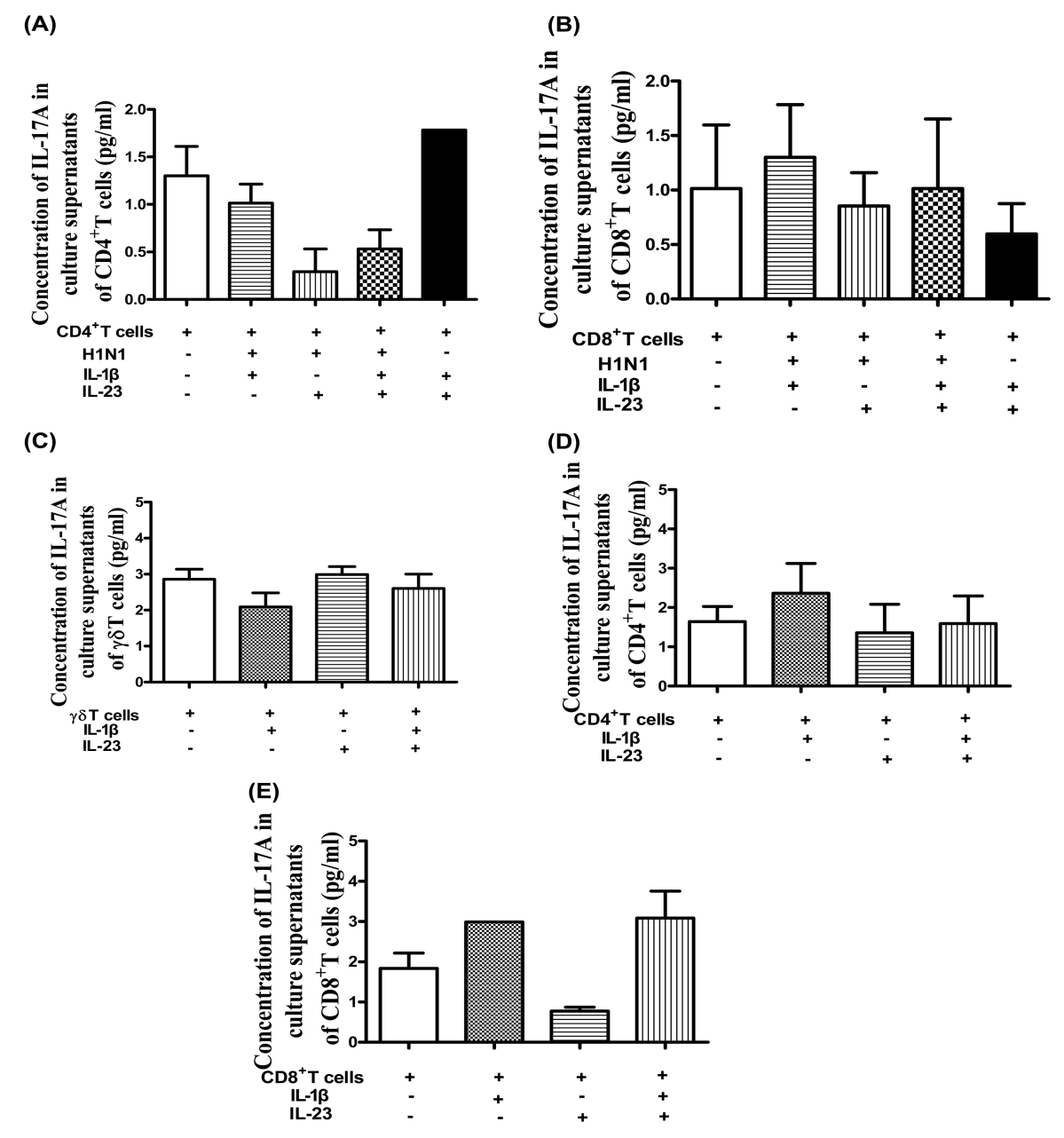


**Supplementary figure 4.** Expression of IL-17A in cultured supernatants of γδT cells, CD4^+^T cells or CD8^+^T cells. MACS-sorted lung-derived CD4^+^T cells (A) or CD8^+^T cells (B) were stimulated with H1N1 virus (10^4^ TCID_50_/100 μl, 5 μl/well), IL-1β (10 ng/ml) and/or IL-23 (10 ng/ml) for 36 h, as indicated. Concentrations of IL-17A in the supernatants were quantified using ELISA. MACS-sorted infected lung-derived γδT cells (C), CD4^+^T cells (D) or CD8^+^T cells (E) at 1 dpi were cultured with IL-1β (10 ng/ml) and/or IL-23 (10 ng/ml) for 36 h, and concentrations of IL-17A in the supernatants were quantified using ELISA.


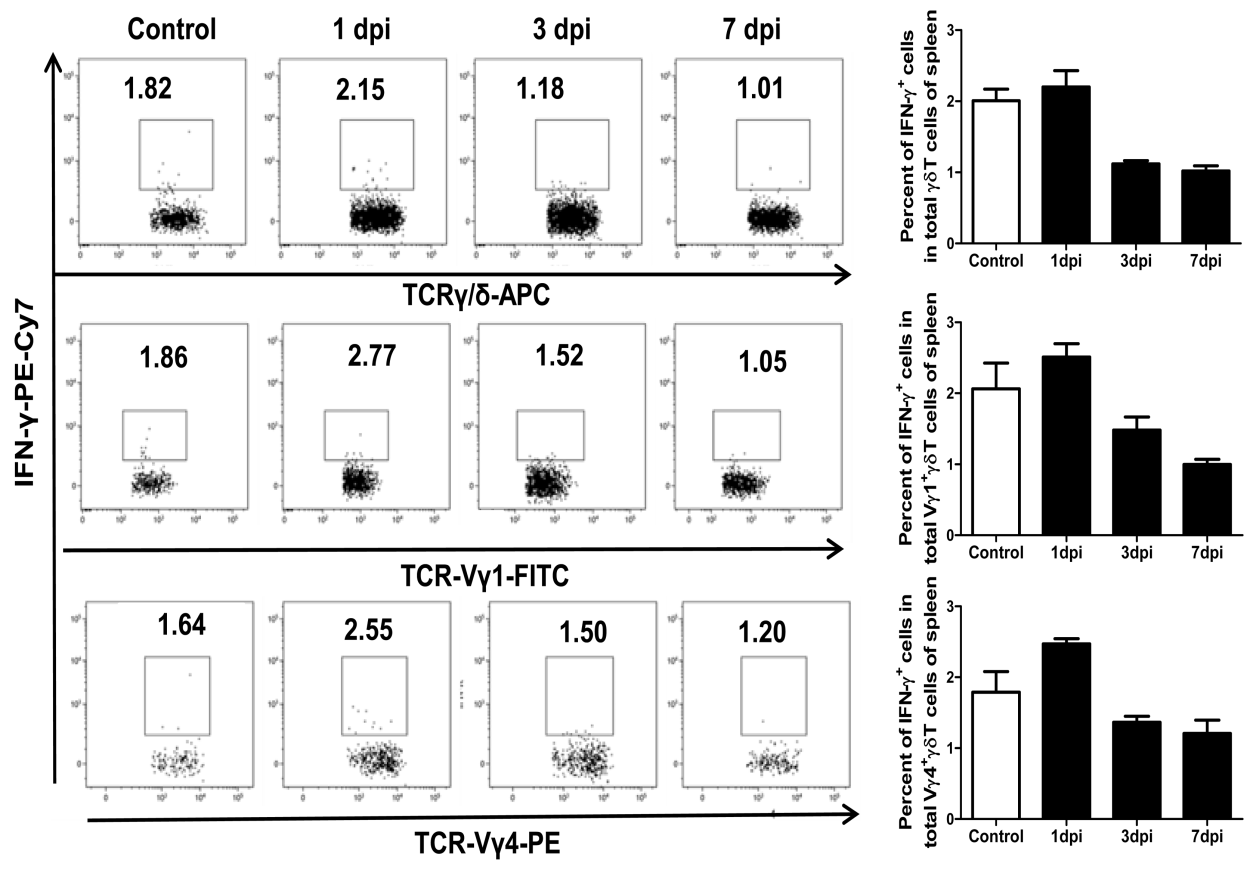


**Supplementary figure 5.** Expression of IFN-γ by γδT cells and Vγ1^+^γδT or Vγ4^+^γδT subsets at different dpi in spleens. The stained spleen lymphocytes were first gated on CD3^+^T cells, and then analyzed the expression of IL-17A in γδT (upper) cells in control or infected spleens at indicated dpi. The expression of IL-17A in Vγ1^+^γδT (middle) and Vγ4^+^γδT (below) subsets were first gated on CD3^+^γδTCR^+^ cells. The number represented the ratio of IL-17A positive cells in different subsets at indicated dpi. Histogram showed the statistical analysis of ratio of different subsets in control or infected spleen at different dpi. The results were representative of two independent studies (n=3 for each group).


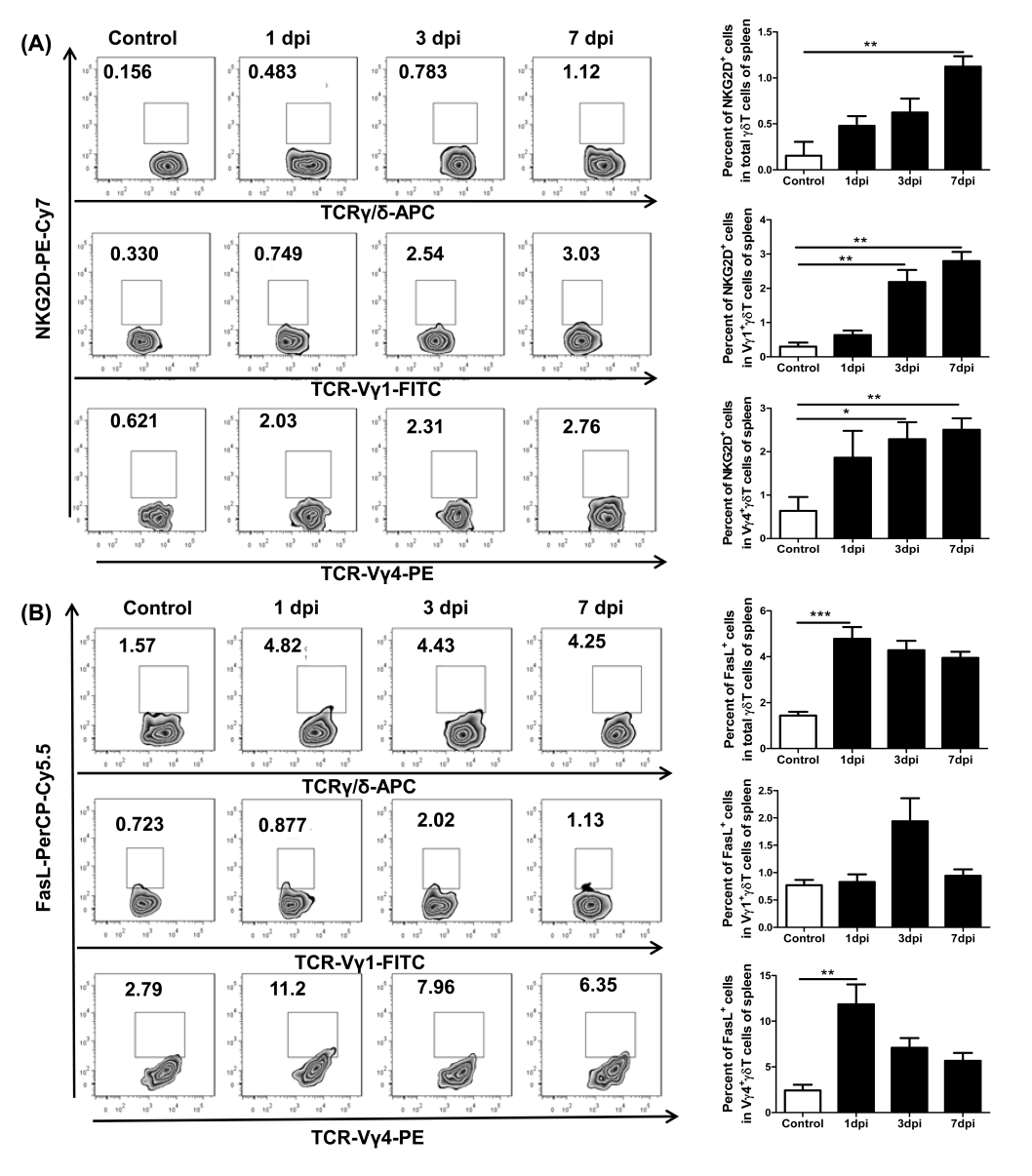


**Supplementary figure 6.** Expression of NKG2D and FasL in γδT cells and Vγ1^+^γδT or Vγ4^+^γδT subsets at different dpi in spleens. The expression of NKG2D (A) and FasL (B) by γδT cells (upper, gated on CD3^+^T cells) and Vγ1^+^γδT (middle, gated on CD3^+^γδTCR^+^ cells) or Vγ4^+^γδT (below, gated on CD3^+^γδTCR^+^ cells) subsets were measured by flow cytometry. The number represented the ratio of NKG2D or FasL positive cells in different subsets at indicated dpi. Histogram showed the statistical analysis of the ratio of NKG2D^+^ or FasL^+^ cells in different subsets in control or infected spleens at different dpi. The results were representative of two independent studies (n=3). The statistical analysis was showed as **P*<0.05, ***P*<0.01, ****P*<0.001.


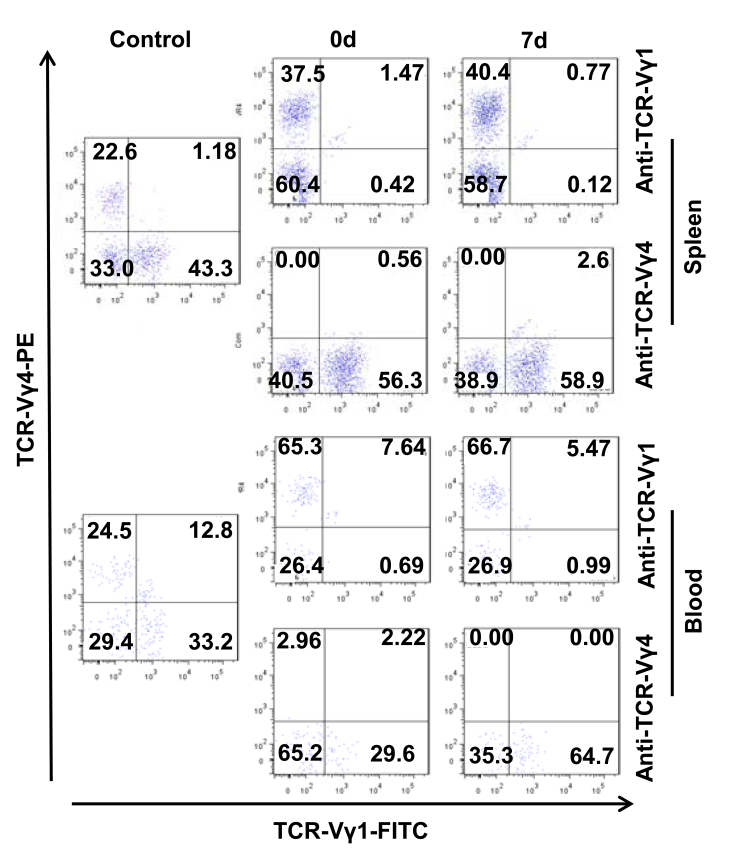


**Supplementary figure 7.** Depletion of Vγ1^+^γδT or Vγ4^+^γδT subsets in spleens and peripheral blood. The depletion of spleens and blood Vγ1^+^γδT or Vγ4^+^γδT subsets (gated on spleens CD3^+^γδTCR^+^ lymphocytes) was detected by flow cytometry at indicated time. The number represented the ratio of different subsets after depletion. The results were representative of two independent studies (n=3 for each group).


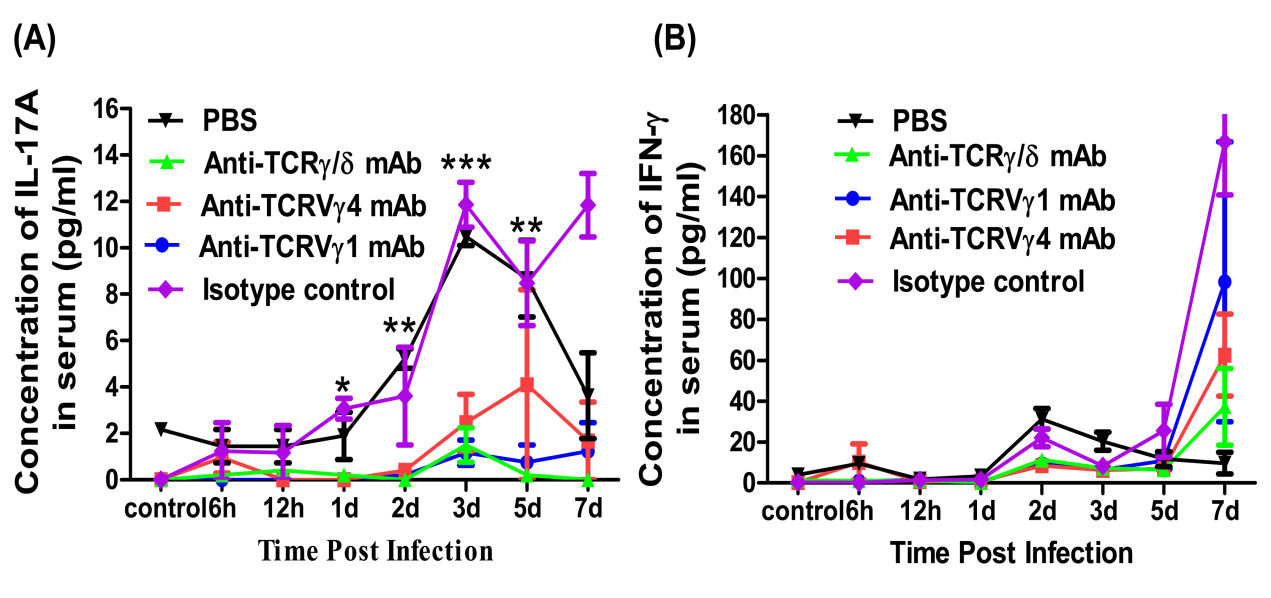


**Supplementary figure 8.** Reduced IL-17A secretion in serum of different cell depletion groups. Concentration of IL-17 (A) and IFN-γ (B) in serum from PBS control group, isotype antibody control group, γδT cell depletion group, Vγ1^+^γδT cell depletion group and Vγ4^+^γδT cell depletion group at the indicated times, were quantified using multiplex biometric immunoassays. The statistical analysis compared the concentration of IL-17A between the control group and cell depletion group. **P* <0.05, ***P* <0.01, ****P* <0.001.
